# The candidate proteins associated with keratoconus: A meta-analysis and bioinformatic analysis

**DOI:** 10.1371/journal.pone.0299739

**Published:** 2024-03-14

**Authors:** Ting Song, Jie Song, Jingyi Li, Halima Ben Hilal, Xiaona Li, Pengfei Feng, Weiyi Chen

**Affiliations:** 1 College of Biomedical Engineering, Taiyuan University of Technology, Taiyuan, China; 2 College of Life Sciences, Fujian Agriculture and Forestry University, Fuzhou, China; University at Buffalo Jacobs School of Medicine and Biomedical Sciences: University at Buffalo School of Medicine and Biomedical Sciences, UNITED STATES

## Abstract

**Purpose:**

Keratoconus (KC) is a multifactorial disorder. This study aimed to conduct a systematic meta-analysis to exclusively explore the candidate proteins associated with KC pathogenesis.

**Methods:**

Relevant literature published in the last ten years in Pubmed, Web of Science, Cochrane, and Embase databases were searched. Protein expression data were presented as the standard mean difference (SMD) and 95% confidence intervals (CI). The meta-analysis is registered on PROSPERO, registration number CRD42022332442 and was conducted in accordance with the Preferred Reporting Items for Systematic reviews and Meta-Analyses statement (PRISMA). GO and KEGG enrichment analysis were performed, as well as the miRNAs and chemicals targeting the candidate proteins were predicted. PPI was analyzed to screen the hub proteins, and their expression was verified by RT-qPCR.

**Results:**

A total of 21 studies were included in the meta-analysis, involving 346 normal eyes and 493 KC eyes. 18 deregulated proteins with significant SMD values were subjected to further analysis. In which, 7 proteins were up-regulated in KC compared with normal controls, including IL6 (SMD 1.54, 95%CI [0.85, 2.24]), IL1B (SMD 2.07, 95%CI [0.98, 3.16]), TNF (SMD 2.1, 95%CI [0.24, 3.96]), and MMP9 (SMD 1.96, 95%CI [0.68, 3.24]). While 11 proteins were down-regulated in KC including LOX (SMD 2.54, 95%CI [-4.51, -0.57]). GO and KEGG analysis showed that the deregulated proteins were involved in inflammation, extracellular matrix (ECM) remodeling, and apoptosis. MMP9, IL6, LOX, TNF, and IL1B were regarded as hub proteins according to the PPI analysis, and their transcription changes in stromal fibroblasts of KC were consistent with the results of the meta-analysis. Moreover, 10 miRNAs and two natural polyphenols interacting with hub proteins were identified.

**Conclusion:**

This study obtained 18 candidate proteins and demonstrated altered cytokine profiles, ECM remodeling, and apoptosis in KC patients through meta-analysis and bioinformatic analysis. It will provide biomarkers for further understanding of KC pathogenesis, and potential therapeutic targets for the drug treatment of KC.

## Introduction

Keratoconus (KC) is a common but serious ophthalmic disease. The prevalence of KC ranges from 1:500 to 1:2000 according to the geographical region [1]. KC is a bilateral progressive corneal ectasia characterized by corneal thinning and protrusion, with impaired visual acuity (myopia and irregular astigmatism), and even blindness [2]. Patients usually exhibit corneal stromal haze in late stage [3,4]. At present, mild and moderate KC patients are usually treated with contact lenses, and corneal cross-linking surgery or corneal transplantation is also applied depending on the severity of KC in the clinic [5].

KC is a multifactorial disorder including genetic and environmental factors [6]. As a chronic injury, eye rubbing is now believed as a risk factor to accelerate the progression of KC [7]. Due to long-term exposure to UV light, the cornea is susceptible to damage by free radicals and reactive oxygen species (ROS), causing an increase in oxidative stress levels [8]. It has also been reported that oxidative damage in KC may be related to mitochondrial dysfunction [9]. Traditionally, KC has been defined as a noninflammatory ectatic corneal disease. However, growing evidence reveals its association with ocular inflammation, particularly increased levels of inflammatory cytokines in the tear fluid of KC [10]. Dynamic changes of inflammatory factors activate metalloproteinases, promote cell apoptosis, and contribute to corneal oxidative damage [11]. A recent study found that the ligand-receptor interaction of anti-inflammatory processes is eliminated in cone cornea [12]. Meanwhile, it also suggested that immune pathways might be involved in the pathogenesis of KC, which has been positively associated with a variety of immune-mediated diseases including allergic rash and asthma [13,14].

As a complex disease, distinguishing the core factors of KC is challenging. Despite lots of studies gain insight into the understanding the fundamental pathogenesis of KC, its underlying mechanism remains unclear. In recent years, gene expression profiles using the whole cornea tissue, epithelium, or stroma layer from individuals with KC have been applied to identify KC-related proteins and pathways using microarray, next-generation sequencing (NGS), and other high-throughput technologies. Dysregulated gene expression has been reported in KC corneal tissue and stromal cells [15–18]. Single-cell RNA sequencing (scRNA-Seq) analysis was performed on 39,214 cells from the central corneas of patients with KC and healthy individuals, confirming the central role of dysregulation of collagen in corneal stromal cells in KC [12]. NGS performed on individual corneal cells from two different patient populations has identified four promising candidate biomarkers and decreased NRF2-antioxidant response in KC disease [17]. Besides, cell migration, adhesion junction, MAPK, Wnt, and Notch1 signaling pathways were involved in KC etiology [16,19]. RNA-Seq-based expression profiling using the whole cornea has identified the downregulation of core elements in TGF-β, Hippo, and Wnt signaling pathways in KC pathogenesis [20]. Integrative omics analyses of KC corneal epithelial tissues and blood samples uncovered a significant association between immune-inflammatory pathways and this disease [21].

Overall, these studies improved our understanding of the molecular mechanisms of KC. Recently, meta-analyses about inflammatory factors and oxidative stress markers in KC have been conducted respectively [22,23]. However, these analyses were limited to one factor in KC pathogenesis. A systematic meta-analysis of this complex disease from the aspect of differentially expressed proteins was lacking. Besides, the reported KC-related proteins varied in different studies because of the varied assay methods and sample sources from study to study. Therefore, it is necessary to perform a meta-analysis to find the candidate proteins of KC pathogenesis. Our meta-analysis summarized the proteins that are disordered in multiple pathogenesis of KC, not limited to inflammatory response or oxidative stress. It will provide candidate proteins involved in KC pathogenesis and potential targets for KC treatment.

## Materials and methods

**Fig 1:**
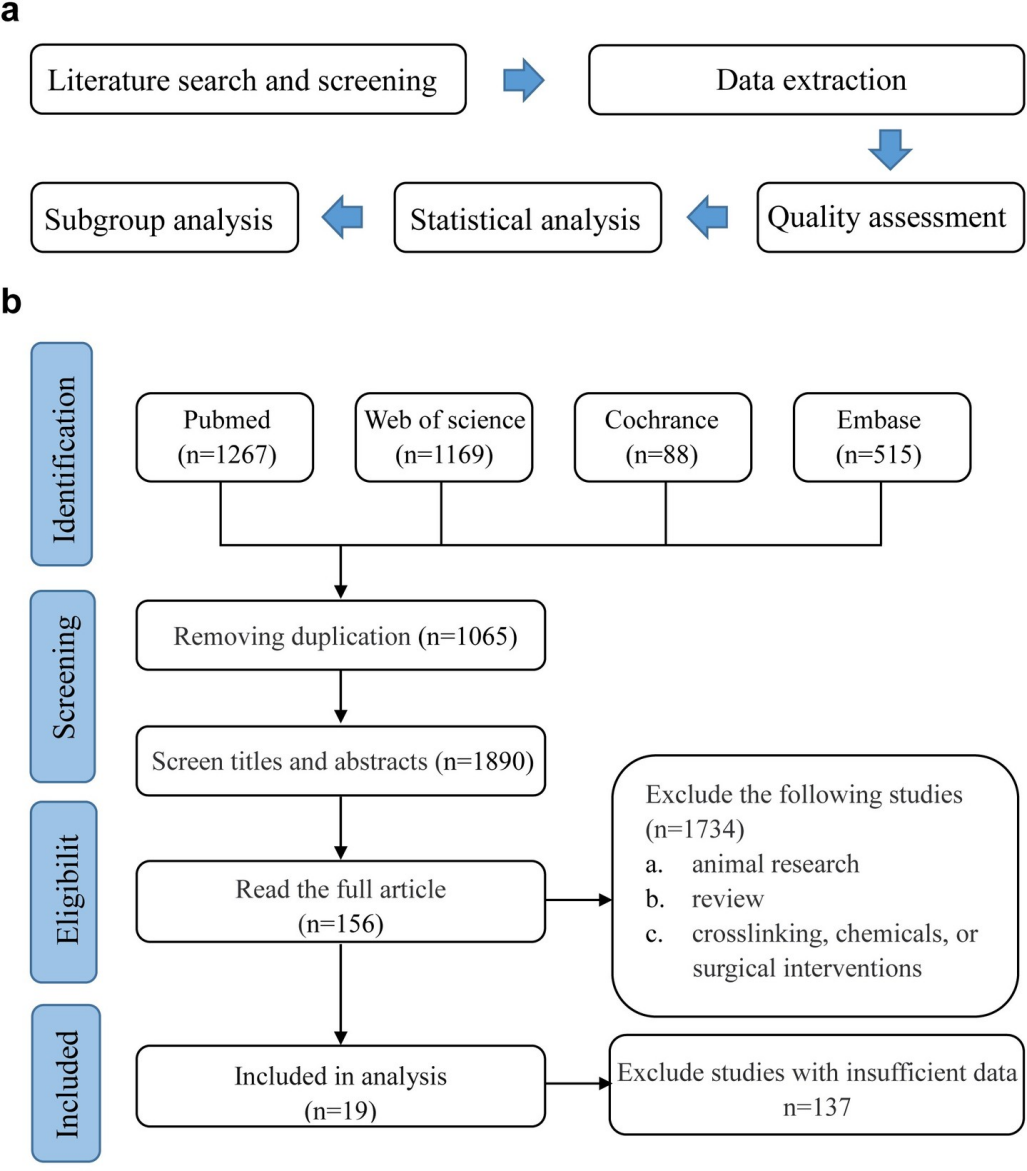
Flow chart of meta-analysis (a) and literature search and selection process (b).

This study is registered in the International Prospective Register of Systematic Reviews (PROSPERO) (registration NO: CRD42022332442) and follows the reporting guidelines of PRISMA 2020 for Systematic Review and Meta-Analysis (S1 Checklist) [24]. The flow chart of this meta-analysis is shown in Fig 1A.

### Search strategy and selection process

Electronic databases including PubMed, Web of science, Cochrane, and Embase were searched for all potential articles published in the past decade (from 1st of January 2012 to 31st of January 2023), with the following keywords “keratoconus” and “proteome” or “transcriptome” or “gene” or “protein”. In addition, references of the included literature were manually searched to identify any other publications not found in the electronic databases. Detailed search strategies for each database are provided in S1 File. Duplicate studies among databases were excluded by NoteExpress software. Studies that did not meet the inclusion criteria were excluded by two independent researchers based on the title and abstract. The remaining studies were screened by reading the full text. Ambiguities that arose were resolved by a third researcher. The selection process is presented in Fig 1B.

### Eligibility criteria for inclusion of articles

Inclusion criteria were as follows: (1) Study of KC pathogenesis at gene or protein level; (2) Human case-control study and the control group must be healthy cornea; (3) English literature. Studies excluded were as follows: (1) Sample size less than 5; (2) Animal research; (3) Reviews; (4) Studies of chemical or surgical interventions; (5) Lack of sufficient data support.

### Data extraction

The data were extracted independently by two researchers from the included studies. The following features were extracted from each study:

1. First author, publication year, and study type.
2. Sample type (tear, cornea, epithelial tissue/cells, or stromal tissue/cells), sample size, country of origin, age and male/female ratio, presence or absence of other ocular diseases in the samples, criteria for determining the disease group and its severity, and the source of the control group and criteria for its selection.
3. The protein being tested, methods of determination (such as PCR, ELISA, mass spectrometry), and protein expression (mean and standard deviation SD).

If more than one different case-controls were reported in the same article, they were treated as independent studies.

### Outcome

The primary outcome included the expression levels of KC related proteins in keratoconus groups compared with healthy controls.

### Quality assessment

The Newcastle-Ottawa Quality Assessment Scale (NOS) was used to score the case–control studies. The following nine items were assessed in 17 studies, including four subjects of selection criteria (a. adequate KC definition; b. representativeness of KC (the course of the disease was accurately graded, and there were no other eye allergic diseases); c. selection of controls; d. definition of controls), two items on comparability between groups (the control group was strictly required to have no family history of KC disease and other allergic diseases), and three items on outcome bias (ascertainment of KC; same method of ascertainment for case and controls; non-response rate). For cross-sectional studies, the Agency for Healthcare Research and Quality (AHRQ) was used to assess 11 items, including (1) Sources of information; (2) Inclusion and exclusion criteria for subjects; (3)Time of identifying patients(KC stage); (4) Consecutiveness of subjects (no significant differences in country, age, or sex of the sample) (5) Interference among outcomes; (6) Assessment for quality assurance; (7) Explain for exclusion; (8) Assess and/or control confounding; (9) Handle the miss data; (10) Response rate of patients; (11) Loss to follow-up. Two authors independently assessed the methodological quality of the included meta-analyses. The disagreements were resolved through a third author.

### Statistical analysis

Statistical analysis was conducted using Stata17 software. Mean and standard deviation (SD) of protein (or gene) expression levels and sample size in KC and controls were summarized for each study. The expression change of proteins is a continuous variable. When similar outcomes were measured using different methods, usually the standardized mean difference (SMD) was calculated to estimate the effects [25]. In this study, we calculated SMD, the difference between the means of the KC group over the control divided by the pooled standard deviation, as the effect value in this study. The effect value above zero (SMD > 0) represented that the protein expression in KC was higher than that in healthy controls. Conversely, the expression in KC was lower than that in healthy controls. Q-statistic (P < 0.05) and I^2^ tests (I^2^ > 50%) were applied to determine heterogeneity. The pooled effects were estimated by a fixed-effect (if I^2^ < 50%, low heterogeneity) or a random-effect (if I^2^ > 50%, substantial heterogeneity) model. Subgroup analysis was used to explore potential sources of heterogeneity.

### Enrichment analysis

The candidate proteins were subjected to Gene Ontology (GO) and Kyoto Encyclopedia of Genes and Genomes (KEGG) enrichment analysis using the R package cluster Profiler. GO items or pathways were considered significantly enriched with a Benjamini-Hochberg adjusted P-value < 0.05.

### MicroRNA(miRNA) prediction and validation for candidate proteins

The miRNAs targeted the candidate proteins were predicted by TargetScan (context scores <-0.2) and miRDB databases (target score >80). mRNA-miRNA network were constructed and visualized using Cytoscape. Afterwards, miRNA expression levels were validated in KC miRNA expression dataset in the GEO database(GSE204838) and visualized by heatmap.

### Protein-protein interaction (PPI) analysis and protein-chemical interactions analysis

To obtain hub proteins for KC pathogenesis, a PPI network of candidate proteins was constructed using the STRING database with a high confidence score of ≥ 900 and requirement for experimental evidence. Then, the PPI network was visualized by Cytoscape, and hub proteins were predicted via CytoHubba [26]. The protein-chemical interactions were analyzed in the Comparative Toxicogenomics Database (CTD) to predict potential drugs that targeted the hub proteins in KC. Chemicals were ranked according to degree and betweenness.

### Isolation and culture of corneal stromal fibroblasts

The present study was conducted in accordance with the Declaration of Helsinki and approved by the Ethics Committee of Taiyuan University of Technology (Taiyuan, China) (No. TYUT 2021090201). Samples of three cases of healthy human cornea and three cases of KC were obtained from Shanxi Eye Hospital (Taiyuan, China). All samples had been obtained between September 1, 2022, and June 30, 2023, and written informed consent was given. All corneas were abandoned tissues after corneal transplantation without other ophthalmic or systemic diseases. The corneal epithelium and endothelium layers were mechanically removed. The remaining corneal stroma was digested with 1 mg/mL of type II collagenase (Worthington Biochemical Corporation) until cells were visible microscopically. Cells were collected by centrifugation and then cultured in DMEM/F12 medium (HyClone) containing 10% fetal bovine serum (FBS, Gibco, ThermoFisher Scientific) at 37°C with 5% CO_2_. Corneal stromal fibroblasts from healthy corneas (HCFs) and keratoconus patients (HKCs) of the third passage were used for RT-qPCR analysis.

### RT-qPCR validation of candidate hub proteins

Total RNA was extracted using an RNA extraction kit (Promega). Reverse transcription was performed using a reverse transcription kit (Takara). Amplification was performed using SYBR qPCR reagent (Takara). The primer sequences are shown in S2 File. The gene expression levels of the target proteins were calculated using 2^(ΔΔCT)^ method. All of the data from three independent experiments were presented as the mean ± SD. Statistical analysis was conducted with SPSS 25.0 software. The statistical significance of the differences between groups was assessed using the independent sample T-test. P<0.05 was considered to indicate a statistically significant difference.

## Results

### Literature search results

In total, 3039 articles were obtained: 1267 in Pubmed, 1169 in Web of science, 88 in Cochrance, and 515 in Embase. After removing 1065 duplicates selected in more than one database and 1890 articles screened by title and abstract (reviews and studies of animal experiments, drugs, surgery, or cross-linked interventions), 156 literatures were remained. Then we performed full-text readings and checked for complete data to calculate effect values SMD. Finally, 19 articles were included for meta-analysis after being reviewed by the inclusion and exclusion criteria. A PRISMA flowchart of the selection process is presented in Fig 1B.

### Characteristics of including studies

Of the 19 literatures included, 4 were cross-sectional studies, and 15 were case-control studies. There were two different case-controls involved in two articles respectively, which were treated as independent studies [21,27]. This resulted in 19 articles describing 21 different studies for inclusion in the following analysis. The key characteristics of the included studies are presented in Table 1. A total of 346 normal control eyes and 493 KC eyes were included in these studies with a sample size of KC group ranging from 3 to 90. The reported ages of KC patients in the included 21 studies ranged from 14 to 44 years old. Eleven of the studies were conducted in Asia [21,27–34] (in which 4 studies from India [29–31,33]), followed by 6 studies from Europe [15,35–39], 2 studies from South America [40,41], and 2 studies from Australia [42,43]. The sample source varied in different studies with whole corneas in 2 studies [15,28], epithelium tissues or cells in 6 studies [21,27,29,30,33,42], stroma tissues in 1 study [27], tears in 10 studies [31,32,34–40,43], and blood in 2 studies [21,41]. More than half of studies (12/21) mentioned the diagnosis of KC according to corneal topography, corneal biomechanics, and slit-lamp examinations by trained corneal specialists. The severity of KC was graded in 14 studies. The expression changes of inflammatory factors, e.g. interleukin 1 alpha (IL1A), interleukin 1 beta (IL1B), interleukin 6 (IL6), tumor necrosis factor (TNF); collagen synthesis proteins and degrading enzymes, e.g. collagen type I (COL I), collagen type IV (COL IV), lysyl oxidase (LOX), matrix metalloproteinase 9 (MMP9), TIMP metallopeptidase inhibitor 1 (TIMP1); immune-related proteins, or proteins in TGF-β, Hippo and Wnt signaling pathways were assessed in KC samples at transcriptional level by RT-PCR in 3 studies or at translation levels using diverse measurement methods including MS (6 studies) and ELISA (5 studies) in other studies (S3 File).

**Table 1:** Study characteristics of all articles included in the meta-analysis.

Studies	Country (area)	Age (KC vs control)	Male/female ratio (KC vs control)	Sample size (KC vs control)	Sample source	Methods of analysis	Proteins
López (2021)[35]	Spain (Europe)	44.88 (5.01) vs 43.96 (6.94)	60% vs 60%	25 vs 25	Tear	LC-MS/MS	HSPB1, FMOD, LTF, et al.
Karolak (2020)[15]	Poland (Europe)	39.83 (8.93) vs 59.17 (15.69)	66% vs 33%	6 vs 6	Corneas	RT-qPCR	LOX
Borges (2020)[40]	Brazil (South America)	30.5 vs 47.5	50% vs 16.67%	4 vs 6	Tear	LC-MS/MS	HSPB1, SFRP1,HPX, LTF, et al.
Xiao Sun (2020)[21]	China (Asia)	16 (2.45) vs 20.14 (1.95)	57.14% vs 57.14%	7 vs 7	Epithelium tissue	RNA-Seq	IL1B,SFRP1, FMOD, et al.
Xiao Sun (2020)[21]	China (Asia)	14.67 (3.06) vs 20 (1.73)	33.33% vs 100%	3 vs 3	Blood	RNA-Seq	LTF, KRT1, et al.
Burcel (2020)[36]	Romania (Europe)	26.13 (8.79) vs 26.57 (9.79)	68.75% vs 64.29%	16 vs 14	Tear	ELISA	ALB
Vishal Shinde (2019)[28]	Saudi Arabia (Asia)	27.6 (5.35) vs 62.4 (5.54)	60% vs 60%	5 vs 5	Corneas	LC-MS/MS	HSPB1, FMOD, HPX et al.
Fai Yam (2018)[27]	Singapore (Asia)	25.3 (5.1) vs 30 (4)	NS	4 vs 2	Epithelium tissue	SWATH-MS	HSPB1, LOX, FMOD, HPX, LTF, et al.
Fai Yam (2018)[27]	Singapore (Asia)	25.3 (5.1) vs 34 (3)	NS	4 vs 2	Stroma tissue	SWATH-MS	HSPB1, FMOD, et al.
Ionescu (2018)[37]	Romania (Europe)	23.35 (11.80) vs 28.66 (3.03)	64.71% vs 40%	17 vs 15	Tear	MILLIPLEX MAP	IL6, IL1B, IL4, TNF, et al.
Tomás Sobrino (2017)[41]	Chile (South America)	33.1 (10.9) vs 30.4 (7.6)	55% vs 55%	40 vs 20	Blood	ELISA	IL1B, IL6, TNF, MMP9, et al.
Natasha Pahuja (2016)[29]	India (Asia)	NS	NS	66 vs 23	Epithelium cell	PCR	IL6, LOX, MMP9, TNF, et al.
Dorottya Pásztor (2016)[38]	Hungary (Europe)	44.2 vs 44.5	NS	55 vs 24	Tear	CBA	IL6,MMP9, et al.
Rohit Shetty (2015)[30]	India (Asia)	22.7 (5.7) vs 22.7 (5.7)	NS	12 vs 10	Epithelial cells	PCR	MMP9, IL6, TNF, et al.
Rohit Shetty (2015)[31]	India (Asia)	28.8 (2) vs 28.0 (3)	27.27% vs 33.33%	7 vs 6	Tear	CBA	IL1B, IL6, IL4, et al.
Priyadarsini (2014)[39]	Denmark (Europe)	30 (10.58) vs 33 (8.58)	NS	17 vs 36	Tear	LC-MS/MS	SFRP1, LTF
Sorkhabi (2014)[32]	Iran (Asia)	24.09 (6.50) vs 24.43 (4.55)	57.14% vs 43.33%	42 vs 30	Tear	ELISA	IL6, IL1B, et al.
Rohit Shetty (2014)[33]	India (Asia)	24.6 (8.4) vs 24.6 (8.4)	NS	90 vs 52	Epithelium cell	ELISA	LOX, MMP9, IL6, et al.
Jingjing You (2013)[42]	Australia (Oceania)	21 (5.8) vs 53 (8.3)	46.67% vs 57.14%	15 vs 7	Epithelium cell	IHC	SFRP1
Jingjing You (2013)[34]	China (Asia)	25.8 (6.7) vs 30.9 (7.5)	57.58% vs 46.88%	33 vs 33	Tear	ELISA	SFRP1
Balasubramanian (2012)[43]	Australia (Oceania)	27.4 (6.0) vs 29.8 (8.9)	NS	25 vs 20	Tear	Antibody Chip	IL6, IL1B, IL4,MMP9, TNF, LTF, et al.

### Quality assessment

The methodological quality of the case–control studies was assessed using NOS and presented in Table 2. Overall, the quality score of the included studies ranged from 6 to 9 points. Most of the studies (78.6%) were of high quality (≥7 points). The remaining studies with 6 points were mainly due to a lack of KC severity grading (6 studies), no rigorous definition of the control group (absence of description of corneal topography, slit-lamp examination, and biomechanical measurements in 9 studies), and no strict exclusion of other ophthalmologic or allergic disorders, contact lens wearing and ocular surgery (13 studies). The methodological quality of the cross-sectional studies was assessed using AHRQ and presented in Table 3. All 4 cross-sectional studies were of high quality (≥9 points). The reason for the loss of score was the significant difference in age and gender between the KC patients and the control group.

**Table 2:** Newcastle–Ottawa Quality Assessment Scale of the case–control studies.

Studies	Selection				Comparability	Exposure			Score
	Adequacy of case definition	Representativeness of case	Selection of controls	Definition of controls	Comparability of groups^a^	Assessment of exposure	Methods of ascertainment	Nonresponse rate	
Karolak(2020)[15]	★		★		★	★	★	★	6
Borges(2020)[40]	★		★	★	★	★	★	★	7
Xiao Sun (2020)[21]	★	★	★		★★	★	★	★	8
Xiao Sun (2020)[21]	★	★	★		★★	★	★	★	8
Burcel(2020)[36]	★	★	★	★	★	★	★	★	9
Vishal Shinde(2019)[28]	★	★	★		★	★	★	★	7
Fai Yam(2018)[27]	★		★	★	★	★	★	★	7
Fai Yam(2018)[27]	★		★	★	★	★	★	★	7
Natasha Pahuja(2016)[29]	★	★	★	★	★★	★	★	★	9
Rohit Shetty(2015)[30]	★	★	★		★	★	★	★	7
Rohit Shetty(2015)[31]	★	★	★		★★	★	★	★	8
Priyadarsini(2014)[39]	★		★		★	★	★	★	6
Sorkhabi(2014)[32]	★	★	★	★	★	★	★	★	8
Rohit Shetty(2014)[33]	★	★	★	★	★	★	★	★	8
Jingjing You(2013)[42]	★	★	★		★	★	★	★	7
Jingjing You(2013)[34]	★	★	★		★	★	★	★	7
Balasubramanian(2012)[43]	★			★	★	★	★	★	6

For each item, meet the standard is: ★, do not meet is none. a: 2 subitems.

**Table 3:** Agency for Healthcare Research and Quality of the cross-sectional studies.

Study	Source of information	Inclusion and exclusion criteria of subjects	Time of identifying patients	Consecutiveness of subjects	Interference among outcomes	Assessment for quality assurance	Explain for exclusion	Assess and/or control confounding	Handle the miss data	Response rate of patients	Loss to follow up	Score
López (2021)[35]	**Y**	**Y**	**Y**	**Y**	**Y**	**Y**	**Y**	**U**	**Y**	**Y**	**Y**	**10**
Ionescu (2018)[37]	**Y**	**Y**	**Y**	**N**	**Y**	**Y**	**Y**	**Y**	**Y**	**Y**	**Y**	**10**
Sobrino (2017)[41]	**Y**	**Y**	**Y**	**N**	**Y**	**Y**	**Y**	**Y**	**Y**	**Y**	**Y**	**10**
Pásztor (2016)[38]	**Y**	**Y**	**N**	**N**	**Y**	**Y**	**Y**	**Y**	**Y**	**Y**	**Y**	**9**

For each item, meet the standard: N (no): 0; Y (yes): 1; U (unclear): 0.

### Meta-analysis of differentially expressed proteins in KC

**Fig 2:**
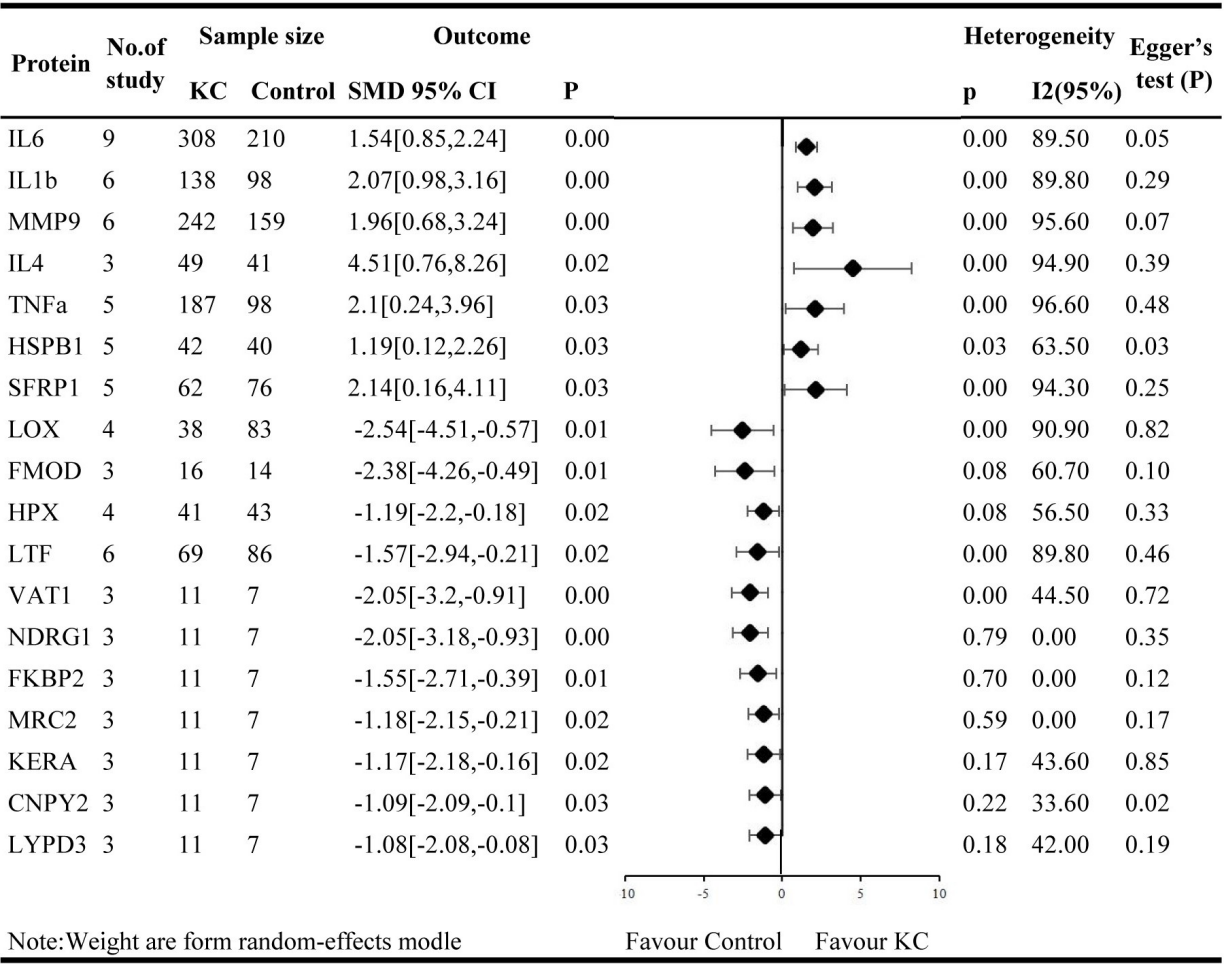
Forest plot of candidate proteins of KC.

This meta-analysis conducted on the differentially expressed proteins between KC and the healthy controls, investigated in at least three independent studies. More than 202 proteins were reported in three or more distinct studies. Among them, 18 proteins expressed significantly differently between KC and normal controls and were regarded as candidate KC proteins for further analysis. Differential expression information for the 18 candidate genes/proteins was supplemented in detail in S4 File. As shown by forest plots in Fig 2, the expression of IL6, IL1B, interleukin 4 (IL4), MMP9, TNF, heat shock protein family B member 1 (HSPB1), and secreted frizzled related protein 1 (SFRP1) were upregulated in KC, while the expression of LOX, fibromodulin (FMOD), hemopexin (HPX), lactoferrin (LTF), vesicle amine transport 1 (VAT1), n-myc downstream regulated 1 (NDRG1), FKBP prolyl isomerase 2 (FKBP2), mannose receptor C type 2 (MRC2), keratocan (KERA), canopy FGF signaling regulator 2 (CNPY2), and LY6/PLAUR domain containing 3 (LYPD3) were downregulated in KC. Other proteins reported in less than three studies or without significant differences were provided in S5 File. In our meta-analyses, we observed high heterogeneity (*I*^*2*^> 50%) for 11 of 18 proteins, indicating variability among studies and associations (Fig 2). For each of the 11 proteins, we conducted further subgroup analysis to discuss the potential factors causing heterogeneity.

The high heterogeneity may partly be due to various sample sources (tear fluids, corneal stroma, and epithelium), different races (Asia, Europe, etc.), and measurement methods (ELISA, PCR, etc.).

**Fig 3:**
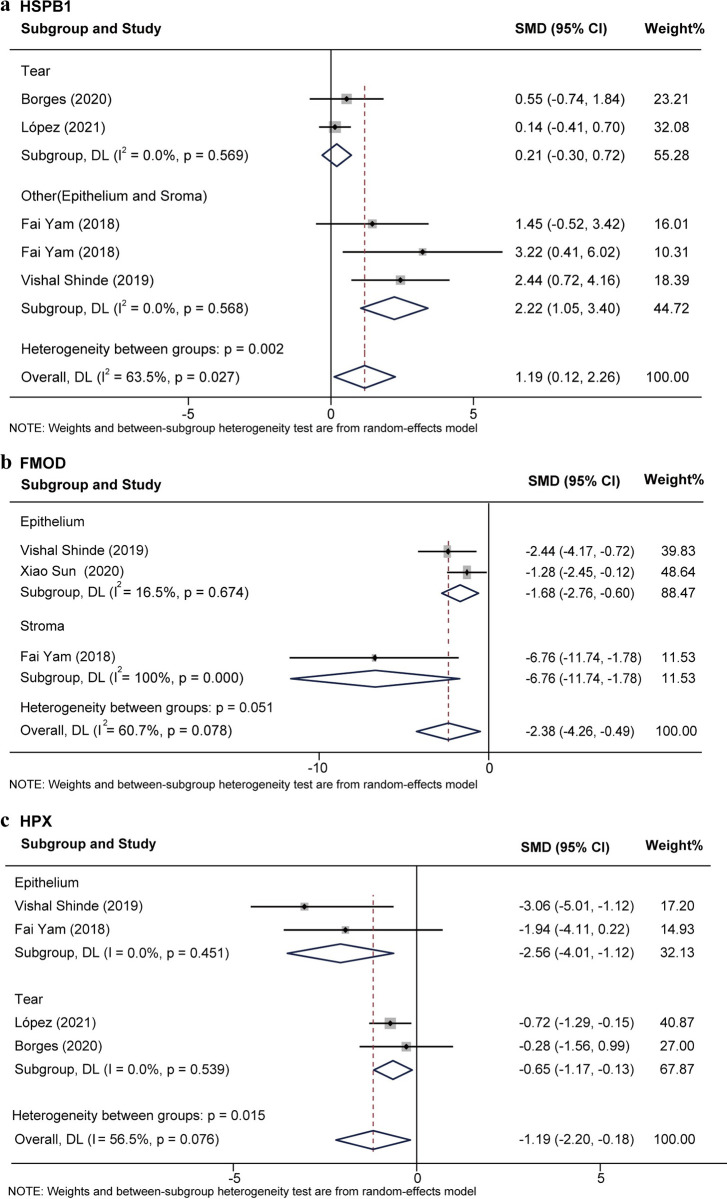
Subgroup analysis of HSPB1, FMOD, and HPX based on sample source.

Subgroup analyses based on sample source (tear fluids, corneal stroma, and epithelium) showed reduced heterogeneity of HSPB1, FMOD, and HPX (S1–S4 Figs). Five studies [27,28,35,40] reported the expression of HSPB1 in total of 42 KC eyes and 40 normal eyes. The total effect value SMD for HSPB1 was 1.19 (95%CI [0.12, 2.26]; *P* = 0.03; *I*^*2*^ = 63.5%), indicating its slightly elevated expression in KC. HSPB1 expression was unchanged in tears (SMD = 0.21; 95%CI [-0.30,0. 72]; *P* = 0.57; *I*^*2*^ = 0) but significantly elevated in corneal epithelium and stroma (SMD = 2.22; 95%CI [1.05, 3.40]; *P* = 0.57; *I*^*2*^ = 0) (Fig 3A). Three studies [21,27,28] reported FMOD expression levels in 16 KC eyes and 14 normal corneal eyes. The SMD value was -2.38 (95%CI [-4.26, -0.49]; *P* = 0.07; *I*^*2*^ = 60.7%). Subgroup analyses showed that FMOD expression levels were reduced and generally less heterogeneous in KC epithelium (SMD = -1.68; 95%CI [-2.76, -0.60]; *P* = 0.27; *I*^*2*^ = 16.5%) (Fig 3B). Four studies [27,28,35,40] reported HPX expression levels in a total of 41 KC eyes and 43 normal eyes. The SMD value was -1.19 (95%CI [-2.20, -0.18]; *P* = 0.08; *I*^*2*^ = 56.5%). Subgroup analyses for sample sources showed significant relevant and generally low heterogeneity with higher SMD in epithelium (SMD = -2.56; 95%CI [-4.01, -1.12]; *P* = 0.45; *I*^*2*^ = 0) and slightly lower SMD in tears (SMD = -0.65; 95%CI [-1.17, -0.13]; *P* = 0.54; *I*^*2*^ = 0) (Fig 3C).

**Fig 4:**
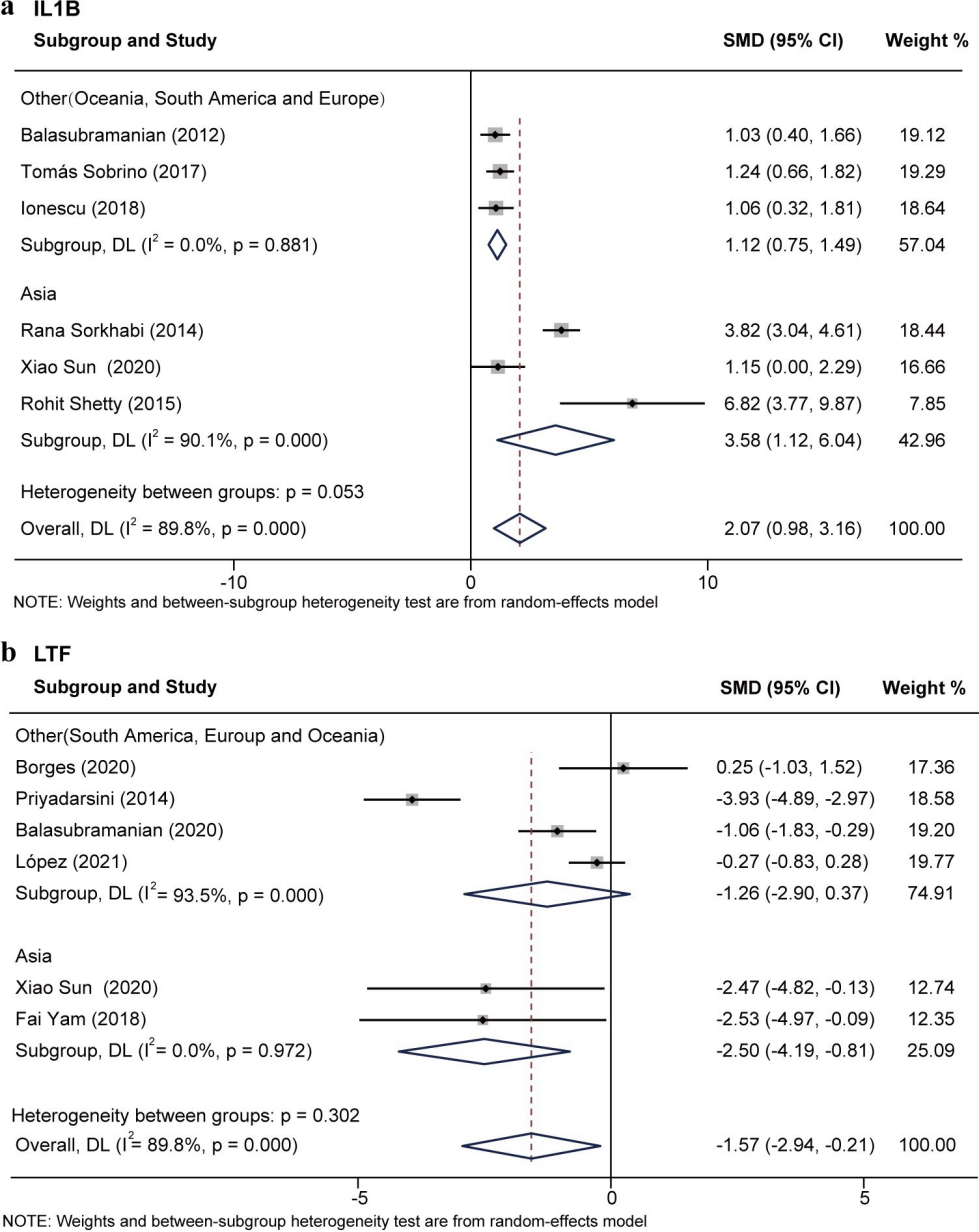
Subgroup analysis of IL1B and LTF based on race.

Subgroup analyses based on different races (Asia, Europe, etc.) showed reduced heterogeneity of IL1B and LTF (S5–S8 Figs). Six studies [21,31,32,37,41,43], including 138 KC eyes and 98 normal eyes, compared the expression of IL1B. The SMD value was 2.07 (95%CI [0.98, 0.16]; P<0.01; I2 = 89.8%) indicating that the expression level of IL1B was elevated in KC. Subgroup analysis showed that IL1B expression was elevated in KC of non-Asian populations and had low heterogeneity (SMD = 1.12; 95%CI [0.75, 1.49]; P = 0.88; I2 = 0). KC expression was also elevated in Asian populations but with high heterogeneity (SMD = 3.58; 95%CI [1.12,6.04]; P<0.01; I2 = 90.1%) (Fig 4A). Six studies [21,27,35,36,39,40] reported LTF expression in 69 KC eyes and 86 normal eyes. SMD value was -1.57 (95%CI [-2.94, -0.21]; P<0.01; I2 = 89.8%). LTF expression was reduced in KC. Subgroup analyses showed that LTF was low in KC in Asian populations with low heterogeneity (SMD = -2.50; 95%CI [-2.94, -0.21]; P = 0.97; I2 = 0). No significant difference of LTF expression in KC of non-Asian populations (SMD = -1.26; 95%CI [-2.90, 0.37]; P<0.01; I2 = 93.5%) (Fig 4B).

**Fig 5:**
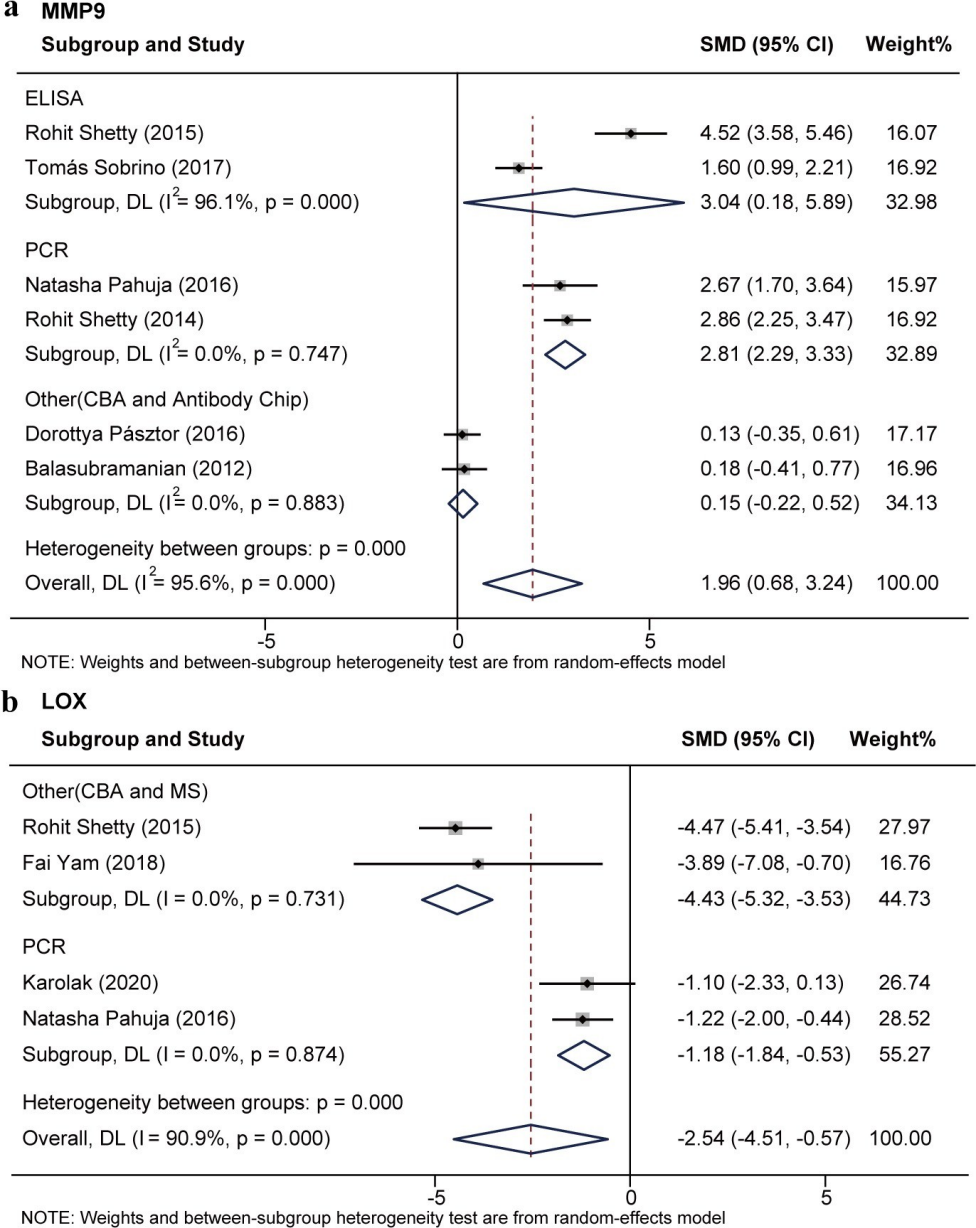
Subgroup analysis of MMP9 and LOX based on measurement methods.

Subgroup analyses based on measurement methods (ELISA, PCR, etc.) showed reduced heterogeneity of MMP9 and LOX (S9–S11 Figs). Six studies [29,30,33,38,41,43] reported expression change of MMP9 between 242 KC eyes and 159 normal eyes. The SMD value for MMP9 was 1.96 (95%CI [0.68, 3.24]; P<0.01; I2 = 95.6%), indicating MMP9 expression was elevated in KC. Subgroup analysis showed that MMP9 was significantly elevated in KC detected by ELISA (SMD = 3.04; 95%CI [0.18, 5.89]; P<0.01; I2 = 96.1%). Its expression was also significantly elevated in KC by PCR analysis (SMD = 2.81; 95%CI [2.29,3.33]; P = 0.75; I2 = 0). No difference was found in MMP9 expression detected using other methods (CBA and Antibody Chip) (SMD = 0.15; 95%CI [-0.22,0.52]; P = 0.88; I2 = 0) (Fig 5A). Four studies [15,27,29,33] reported LOX expression levels in a total of 38 KC eyes and 83 normal corneal eyes. The SMD value was -2.54 (95%CI [-4.51, -0.57]; P<0.01; I2 = 90.9%). Subgroup analyses showed a significant reduction and low heterogeneity of LOX in KC detected using PCR (SMD = -1.18; 95%CI [-1.84, -0.53]; P = 0.87; I2 = 0) and other methods (CBA and MS) (SMD = -4.43; 95%CI [-5.32, -3.53]; P = 0.73; I2 = 0) (Fig 5B).

Nine studies [29–33,37,38,41,43], including a total of 308 KC eyes compared with 210 healthy control eyes, investigated the expression changes of IL6. The SMD value was 1.54 (95%CI [0.85, 2.24]; P<0.01; I2 = 89.5%). Three studies [31,37,43] have collectively examined IL4 expression in tears of 49 KC eyes and 41 normal eyes. SMD value was 4.51 (95%CI [0.76, 8.26]; P<0.01; I2 = 94.9%). Five studies [29,30,37,41,43] reported TNF expression levels in 187 KC eyes and 98 normal eyes. The SMD value was 2.10 (95%CI [0.14, 3.96]; P<0.01; I2 = 96.6%). Five studies [21,34,39,40,42] reported SFRP1 expression in a total of 42 KC eyes 41 normal eyes with an SMD value of 2.14 (95%CI [0.16, 4.11]; P<0.01; I2 = 94.3%),which was elevated in KC. All four of the above proteins have high heterogeneity. Subgroup analysis showed that the high heterogeneity of IL6, IL4, TNF, and SFRP1 could not be explained by different sample sources, races, or methods. Varied KC stage, differences in sample sizes (range of 4–90 KC eyes and 2–52 healthy eyes), and matching of age and sex also potentially contributed to the heterogeneity observed.

### Enrichment analysis of candidate KC proteins

**Fig 6:**
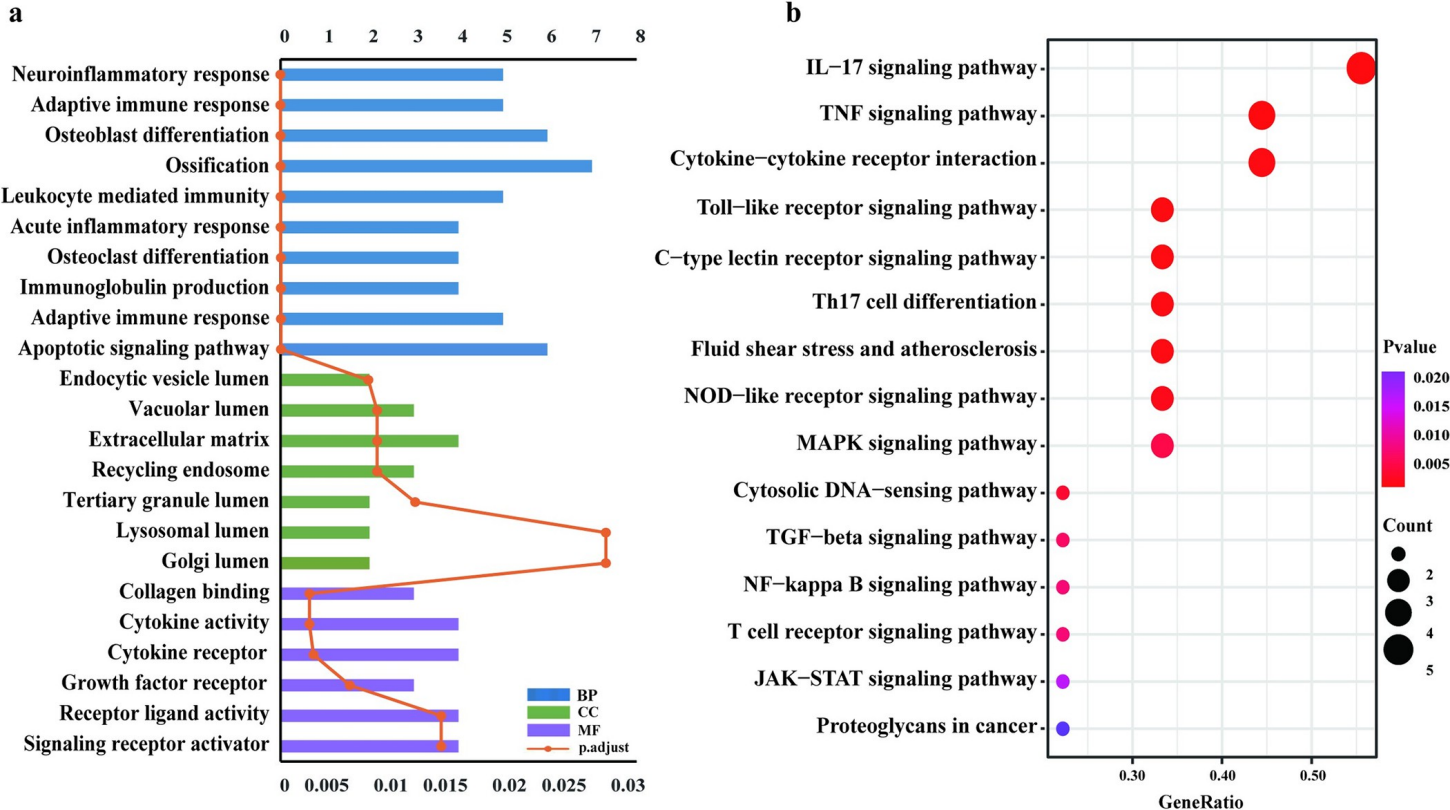
Enrichment analysis of the candidate KC proteins. (a) GO enrichment analysis. The three colors represent biological processes (blue), cell components (green), and molecular function (purple), respectively. (b) KEGG pathway enrichment analysis.

GO analysis showed that candidate KC proteins enriched in 758 biological processes, 7 cell components, and 6 molecular function terms (p < 0.05). The main significant enriched biological process and molecular function were associated with ossification and apoptotic signaling pathway, and collagen binding and growth factor receptor binding, respectively (Fig 6A). Besides, the enriched cellular component was extracellular matrix (ECM) (Fig 6A). GO analysis of candidate KC proteins indicated the involvement of inflammation, matrix remodeling, and apoptosis in keratoconus pathogenicity. KEGG pathway enrichment analysis showed that the candidate KC proteins obtained by meta-analysis were mainly involved in the IL-17 signaling pathway, TNF signaling pathway, MAPK signaling pathway, and TGF-beta signaling pathway (Fig 6B). Thus, combining the enrichment analysis and reported functions in the literature, the 18 candidate KC proteins were categorized: inflammatory cytokines (IL6, IL1B, IL4, MMP9, and TNF), ECM-related proteins (LOX, MRC2, FMOD, and KERA), oxidative stress proteins (HSPB1, HPX, LTF), apoptosis-related protein SFRP1, and others (VAT1, NDRG1, FKBP2, CNPY2, LYPD3).

### PPI network of candidate KC proteins and gene expression analysis in KC stromal fibroblasts

**Fig 7:**
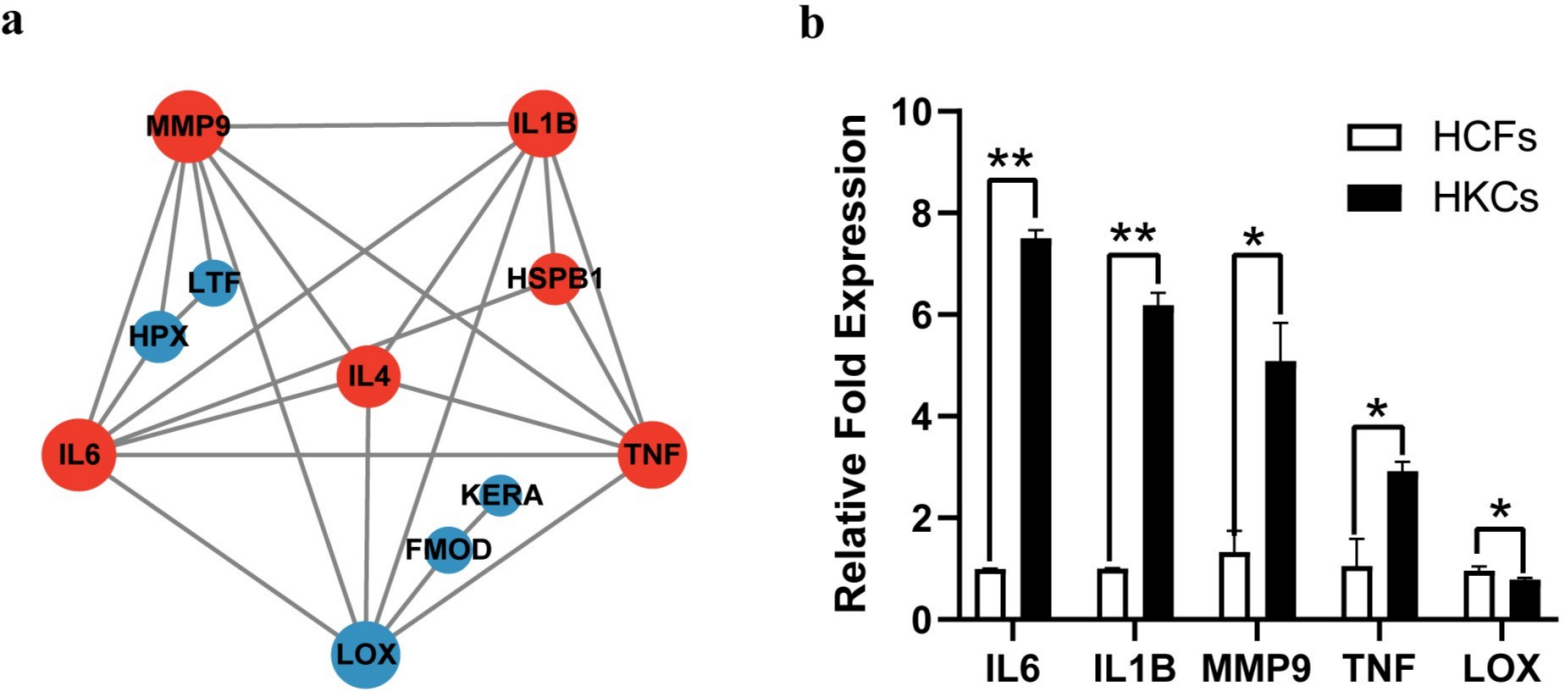
PPI network of candidate KC proteins (a). Gene expression changes in KC stromal fibroblasts (b).

The PPI network of candidate KC proteins with 11 nodes and 24 connection edges was constructed as exhibited in Fig 7A. On this basis, the 5 proteins including MMP9, IL6, LOX, TNF, and IL1B were regarded as hub proteins for KC according to the node degrees (degrees≥6). Furthermore, RT-qPCR was used to verify the transcription levels of the five hub proteins in KC stromal cells. Compared with the healthy controls, the gene expression levels of *MMP9*, *IL6*, *IL1B*, and *TNF* significantly increased in KC stromal, while the gene expression of *LOX* decreased (Fig 7B). The expression changes of the hub proteins in KC were consistent with the results of the meta-analysis in Fig 2.

### Construction and analysis of mRNA-miRNA network

**Fig 8:**
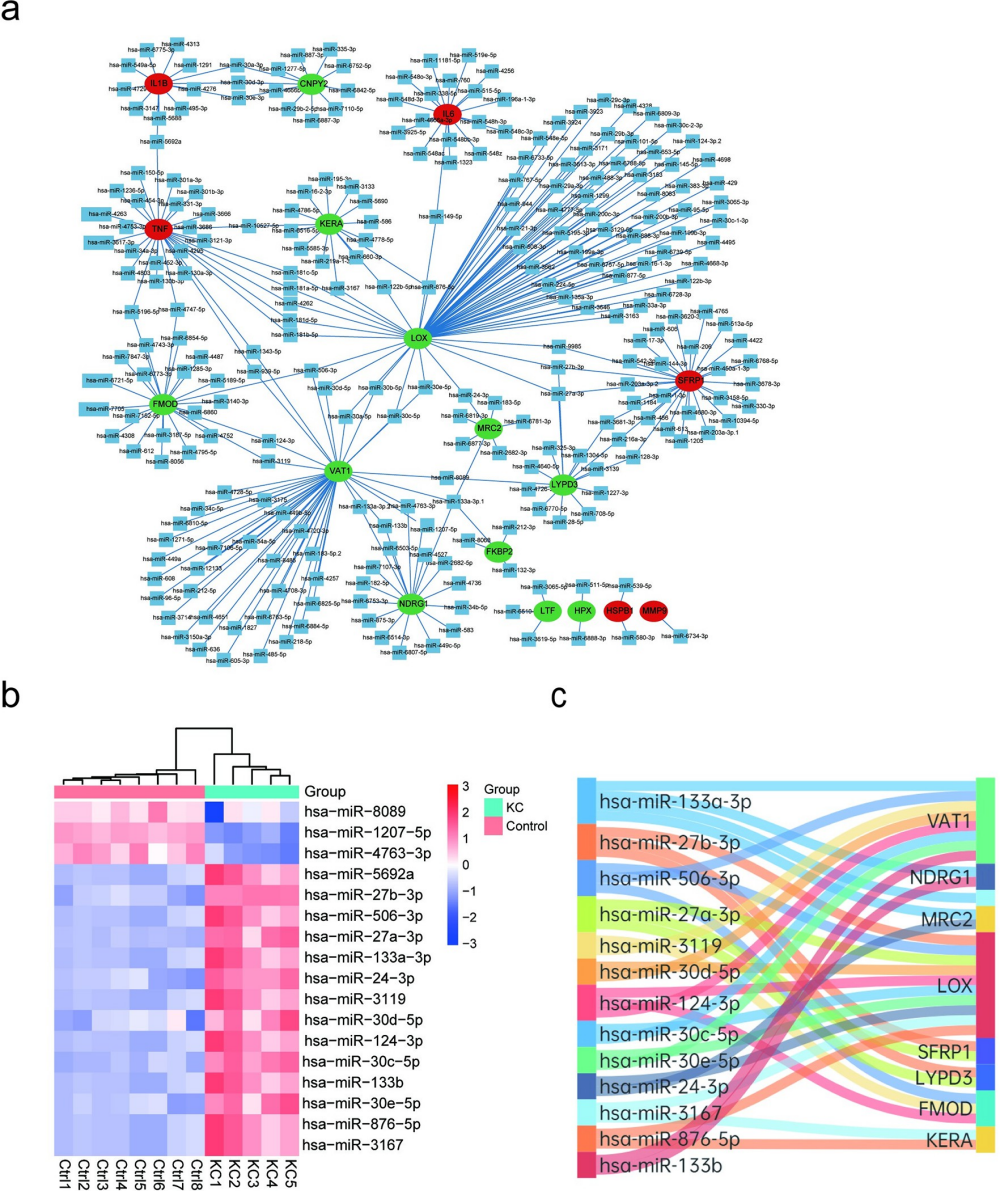
miRNA-mRNA interaction analysis for candidate KC proteins. (a) miRNA-mRNA network. Red means up-regulated, green means down-regulated, and blue means miRNA.(b) Expression changes of miRNAs in KC by a heatmap plot. The higher expression was colored in red, and the lower expression was colored in blue. (c) Sankey diagram for the miRNA-mRNA network.

miRNAs targeted the candidate KC proteins were predicted and the mRNA-miRNA network with 281 nodes and 607 edges was presented in Fig 8A. Among them, we screened 20 miRNAs that can target two or more candidate proteins with consistent expression changes in KC. Furthermore, the expression changes of these 20 miRNAs in KC were validated in the GEO database (Fig 8B). Finally, 13 key miRNAs with opposite expression changes to their target proteins in KC were obtained (Fig 8C). These 13 miRNAs targeted 9 candidate proteins, which were listed in the S6 File. In which, 10 miRNAs including miR-27b-3p, miR-506-3p, miR-27a-3p, miR-30d-5p, miR-124-3p, miR-30c-5p, miR-30e-5p, miR-24-3p, miR-3167, and miR-876-5p, target LOX, one of the hub proteins for KC identified in this study.

### Prediction of potential therapeutic drugs for KC

**Fig 9:**
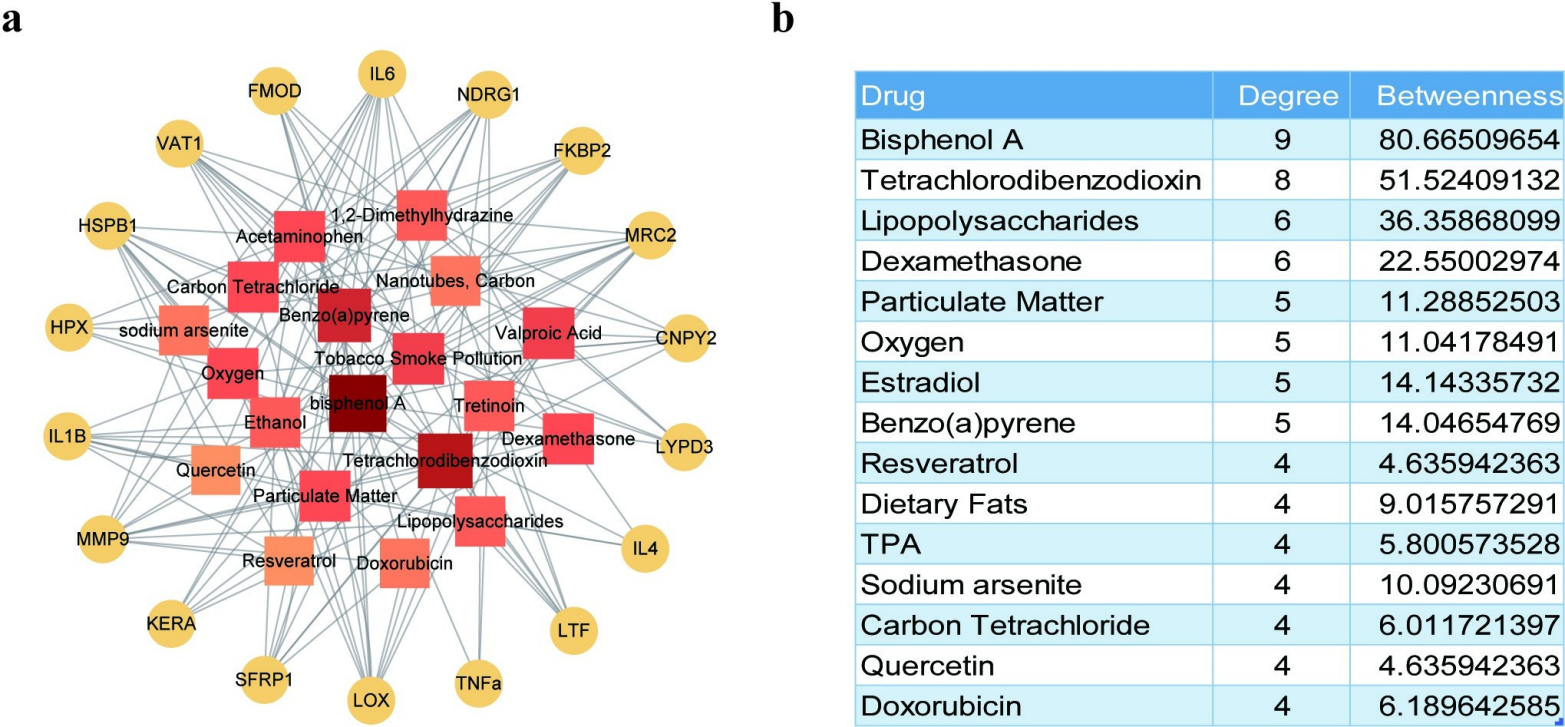
Prediction of potential therapeutic targets for KC. (a) Protein-chemical interaction network. Yellow circles represented candidate KC proteins, and the squares represented the chemicals which were colored according to the degree and betweenness of protein-chemical interactions. (b) The top 15 drugs associated with candidate KC proteins in the protein-chemical interactions network.

The protein–chemical interaction analysis of candidate KC proteins helps to explore the potential therapeutic drugs for KC. A protein-chemical (top 20) interaction network with 193 nodes and 360 edges was constructed (Fig 9A). In total, 175 chemicals were identified to interact with the candidate KC proteins, and the top 15 drugs were listed in Fig 9B. Among the compounds, we found that resveratrol and quercetin, two natural polyphenols, could interact with the hub protein MMP9 in KC.

## Discussion

KC is an ocular disorder with a multifactorial origin. Meta-analysis of inflammatory cytokines, oxidative stress markers, gene polymorphisms, allergic eye diseases, and eye rubbing in KC have been conducted previously [22,23,44–46], indicating the important roles of these factors in KC pathogenesis. In this updated meta-analysis, we aimed to assess the expression levels of disordered proteins in multiple pathogenic mechanisms of KC. Our results identified 18 candidate proteins with significantly different expressions between KC and healthy cornea. Among them, the expression levels of inflammatory factors (e.g., IL1A, IL1B, IL6, TNF), collagen degrading enzyme (MMP9), and apoptosis-related protein (SFRP1) in patients with KC were significantly higher than those of healthy people, while the expression levels of ECM-related proteins (e.g., LOX, MRC2, FMOD, and KERA) were downregulated in KC, indicating the disorders of inflammatory factors, matrix metabolism, and apoptosis in KC. Moreover, 10 miRNAs (miR-27b-3p, miR-506-3p, etc.) and two drugs (resveratrol and quercetin) interacting with the candidate proteins were obtained by bioinformatics analysis. Although KC is traditionally defined as a non-inflammatory corneal ectasia, more and more studies indicate the association between inflammation and KC [10]. A recent meta-analysis discussed the levels of five inflammatory cytokines in KC tears, including IL1B, IL4, IL6, IL10, and TNF [23]. Increased levels of inflammatory factors have also been demonstrated in epithelial and blood samples from KC [30,47], suggesting that the tear microenvironment of KC was in an inflammatory state. KEGG enrichment analysis of the candidate KC proteins in this study also confirmed the involvement of inflammation in KC. Moreover, the expression of TNF increased with the grade of KC and correlated with the degree of corneal deformation [30]. In multiple studies, it has been found that the expression levels of IL1B, IL6, and TNF in the tear fluids or epithelial cells were significantly elevated in KC patients [23,31]. IL4, a central role in atopic diseases, was also an important risk factor for KC [48]. IL10 is another primarily anti-inflammatory cytokine. The concentrations of IL4 and IL10, in the tear film of KC patients and their first-degree family members were significantly higher than those in the control group [37]. Conversely, in some studies, the expression of IL10 was reduced in KC tears or epithelium [29,32]. Results of the previous meta-analysis showed that the levels of IL4 and IL10 didn’t change significantly in KC tears [21]. Our results also showed no significant change of IL10, but significant increase of IL4 expression in KC patients. The reason for this discrepancy may be that the resources in our study not only included tears, but also corneal epithelium, stroma, and blood from KC patients. The role of IL4 in KC still needs to be investigated in further studies.

KC is characterized by thinning and weakening of the corneal stroma, which is the main load-bearing part of the cornea. GO analysis in this study indicated that the candidate KC proteins including MMP9, LOX, MRC2, FMOD, and KERA were involved in ECM remodeling. Collagen-degrading enzymes such as MMP9 and cross-linking enzymes such as LOX were found dysregulated in ocular diseases [33]. The expression levels of LOX and MMP9 were correlated with KC severity and could be used as markers for KC grading [33]. In our study, MMP9 and LOX were regarded as hub proteins for KC. LOX could improve the corneal biomechanical properties. Precious studies focused on the SNPs of LOX, suggesting that LOX variants may affect individual susceptibility to KC [45]. In addition, the expression level of LOX was significantly reduced in corneal epithelium, stroma, and tear fluids of KC patients [15,29,33]. MMPs levels were increased in cornea and tears of KC patients, particularly MMP1 and MMP9, indicating disordered protein hydrolysis in KC [49]. MRC2, also known as uPARAP/Endo180, is essential for cellular uptake of collagen. Endo180-deficient fibroblasts showed complete loss of cellular uptake of collagen types I, IV, and V [50]. FMOD was enriched in the TGF-β signaling pathway and appeared in many KC omics studies [28,51,52]. Our study showed reduced expression of MRC2 and FMOD in the KC, while their functions in KC were rarely reported.

A previous meta-analysis has shown that oxidative stress markers and antioxidants were dysregulated in KC [22] HSPB1 is a negative regulatory factor of ferroptosis and plays an important role in the regulation of intracellular redox status [53] and cell migration [54]. Increased expression of HSPs in ocular diseases (such as cataracts and glaucoma) protects the eye from ocular stress or damage by refolding damaged proteins. LTF is an iron-binding protein that functions in innate and adaptive immune responses. It exhibits antibacterial, anti-inflammatory, and antioxidant activities in tears [55], suggesting its potential as a therapeutic approach for KC. There was a strong positive correlation between LTF and central corneal thickness in KC patients (r = 0.641, p = 0.007) [36]. SFRP1 accumulation may induce apoptosis in corneal epithelial cells [55,56]. In a study of differentially expressed genes (DEGs) in the epithelium and stroma of eight healthy and eight KC corneas, the most upregulated gene in the epithelium was *SFRP1* [18]. In the present study, we also found up-regulation of HSPB1 and SFRP1, with a down-regulation of LTF, confirming the elevated levels of oxidative stress and cell apoptosis in KC.

KC is a disease not only closely related to changes of biomechanical properties of cornea, but also changes of mechanical environment where keratocytes reside in. The corneal biomechanical properties are significantly lower in KC. It has been proposed that the focal weakness is the initiating event in KC, resulting in a cycle of biomechanical decompensation with increased strain and thinning in the weak area, leading to a focal increase in stress [57]. In addition, as a risk factor for KC, vigorous eye rubbing was shown sharply increased force to corneas [58]. In our previous studies, we found that mechanical stretch could induce the expression of the hub genes including IL6 [59]. Moreover, IL6 and MMP9 were positively correlated with maximum keratometry (Kmax) [37,60], a morphological parameter that can reflect deformation degree of the cornea. IL6 and IL1B were significantly negatively correlated with central corneal thickness (CCT) in KC [37]. Corneal cross-linking(CXL) can effectively halt the progression of KC by strengthen collagen fibril linkages and therefore the biomechanical properties of cornea. LOX and MMP9 are two enzymes that regulate the formation and maturation of collagen [61]. Decrease in activity and quantity of LOX in KC patients resulted in cross-linking disorder and weakened biomechanical properties of the cornea [62]. It was reported that the expression and activity of LOX could be restored after CXL [63]. MMP9 may impair the stability of the ECM by decreasing collagen synthesis. After CXL, the expression of MMP9 was reduced, decreasing the inhibitory effect on collagen synthesis [64]. In addition, the expression of TNF was reduced after cross-linking [65]. All these studies provided a evidence for the correlation between the biomechanical properties of cornea and the hub proteins identified in this analysis.

Post-transcriptional regulation by miRNAs has been proven to be involved in many ocular diseases [66]. The role of miRNAs in KC has been explored recently [18,67,68]. Thirteen miRNAs in our study showed up-regulation expression in conical corneas, and their targeted mRNAs were lowly expressed in KC corneas. miR-506-3p targeted three candidate KC proteins including LOX, FMOD, and VAT1; miR-506 was involved in the regulation of differentially expressed proteins (early growth response 1 (EGR1), GA binding protein transcription factor subunit alpha (GABPA), FOS, MYC associated factor X (MAX), JUN) in the tear fluids of thyroid‑associated patients. However, its role in KC remains unclear. Thus, the functions of candidate proteins in KC disease and the regulation mechanism need to be further investigated.

KC treatment varies depending on disease severity. Mild and moderate cases are typically treated with rigid gas permeable lenses (RGPs) and CXL, while severe cases that cannot be managed with RGPs and corneal cross-linking may require corneal transplantation [69]. At present, drug therapy for KC is still limited. A significant reduction of IL6 and TNF at the mRNA level was observed in KC patients treated with cyclosporine A (CyA). In addition, CyA can reduce MMP9 expression in KC through long-term inhibition of inflammatory signaling pathways [30]. Studies have shown that vitamin D (Vit D) supplementation can cause a decreased MMP9 level and an increased TIMP1 expression. Vit D may control the KC progression by improving Cu metabolism, leading to an increase in endogenous collagen cross-linking and antioxidant capacity [70]. Sulforaphane could prevent KC progression by activating the Nrf-2/HO-1 antioxidant pathway in rabbit KC models [71]. Quercetin has been reported to regulate fibrosis in KC by altering cellular metabolism and reducing lactate production [72]. Through the protein-chemical interaction network, we found 15 chemicals including resveratrol and quercetin that can interact with most of the hub KC proteins, providing a new direction for the drug therapy of KC.

### Limitations and prospects

Firstly, due to the diversity of detection methods, direct statistical analysis could not be performed, and many studies were excluded due to a lack of data for calculating SMD values, resulting in the sample size of the original study being limited. Secondly, KC is a multifactorial disease, and various test methods, sample sources, ages, and race of the participant populations in the various studies could also result in its heterogeneity. Although the present meta-analyses for KC cannot avoid suffering from relatively high heterogeneity [22,23,73,74], it is still helpful for understanding the fundamental pathogenesis of this disease. In this study, we use different effects model in our meta-analysis (fixed effects model for low heterogeneity and random effects model for high heterogeneity) [75,76]. Besides, we conducted subgroup analyses to minimize heterogeneity as much as possible.

Due to samples obtaining from different KC stages, the progressive or prognostic markers for this disease were not identified in our current study. However, it was shown that the hub proteins (IL-6, MMP9, TNF, and LOX) identified in this study were correlated with the severity of KC [33,37,77]. Besides, inhibition of MMP9 expression could delay the progression of KC [78]. CXL is a promising treatment for KC and attracts widespread attention in the last decade [79]. The expression and activity of LOX were restored while the expression of MMP9 was reduced in KC after CXL [63,80]. Thus we supposed that the hub proteins MMP9, IL6, LOX and TNF might be the potential biomarkers for KC progression or prognosis. It should be further validated through animal models of KC in our future study. And among the 18 candidate proteins identified in our analysis, the functions of VAT1, NDRG1, FKBP2, CNPY2 and LYPD3 in KC pathogenesis remain unclear. An in-depth study of these candidate proteins may provide a new perspective for KC pathogenesis. It would further indicate some new proteins related to KC progression.

## Conclusions

We obtained 18 KC candidate proteins by meta-analysis, which were mainly involved in inflammatory response, ECM remodeling, and apoptosis. Subgroup analysis yielded different expression patterns of the candidate proteins, which would help to better understand the molecular mechanism of KC pathogenesis. In addition, potential therapeutic drugs targeting the candidate proteins were predicated. This study is a valuable reference for revealing the pathogenesis of KC and its early intervention.

## Supporting information

S1 Checklist PRISMA 2020 checklist. (PDF)

S1 Fig Subgroup analysis of IL6 and IL1B based on sample source. (TIF)

S2 Fig Subgroup analysis of MMP9 and TNF based on sample source. (TIF)

S3 Fig Subgroup analysis of HSPB1, SFRP1 and TNF based on sample source. (TIF)

S4 Fig Subgroup analysis of MMP9 and TNF based on sample source. (TIF)

S5 Fig Subgroup analyses of IL6 and IL1B based on different races. (TIF)

S6 Fig Subgroup analyses of MMP9, TNF and HSPB1 based on different races. (TIF)

S7 Fig Subgroup analyses of SFRP1, LOX and HPX based on different races. (TIF)

S8 Fig Subgroup analyses of LTF based on different races. (TIF)

S9 Fig Subgroup analyses of IL6, IL1B and MMP9 based on measurement methods. (TIF)

S10 Fig Subgroup analyses of TNF, SFRP1 and LOX based on measurement methods. (TIF)

S11 Fig Subgroup analyses of FMOD and LTF based on measurement methods. (TIF)

S1 File Search strategies in PubMed, Web of Science, Cochrane and Embase databases. (PDF)

S2 File Primer sequences for qPCR. (PDF)

S3 File Study characteristics of articles included in the meta-analysis. (PDF)

S4 File Differential expression information for the 18 candidate genes/proteins. (PDF)

S5 File Effect values for proteins (more than three study reports). (PDF)

S6 File The 13 miRNAs targeted 9 candidate proteins. (PDF)
