# Supplementary figures and images for: The candidate proteins associated with keratoconus: A meta-analysis and bioinformatic analysis
Source: PLoS One. 2024 Mar 14;19(3):e0299739. doi: 10.1371/journal.pone.0299739 (PMC10939257; doi:10.1371/journal.pone.0299739)

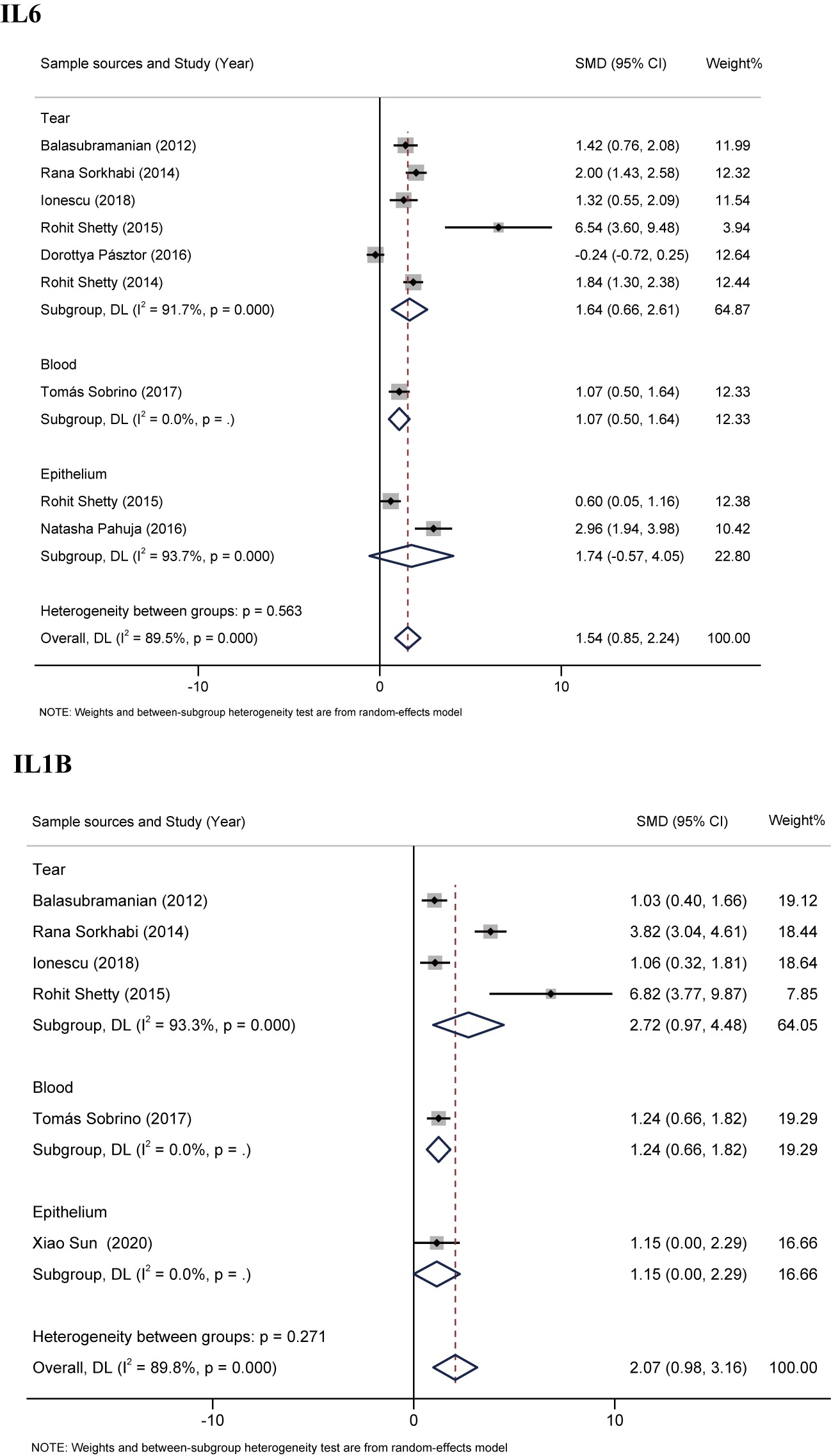

Supplement: S1 Fig — (TIF) [file pone.0299739.s002.tif]

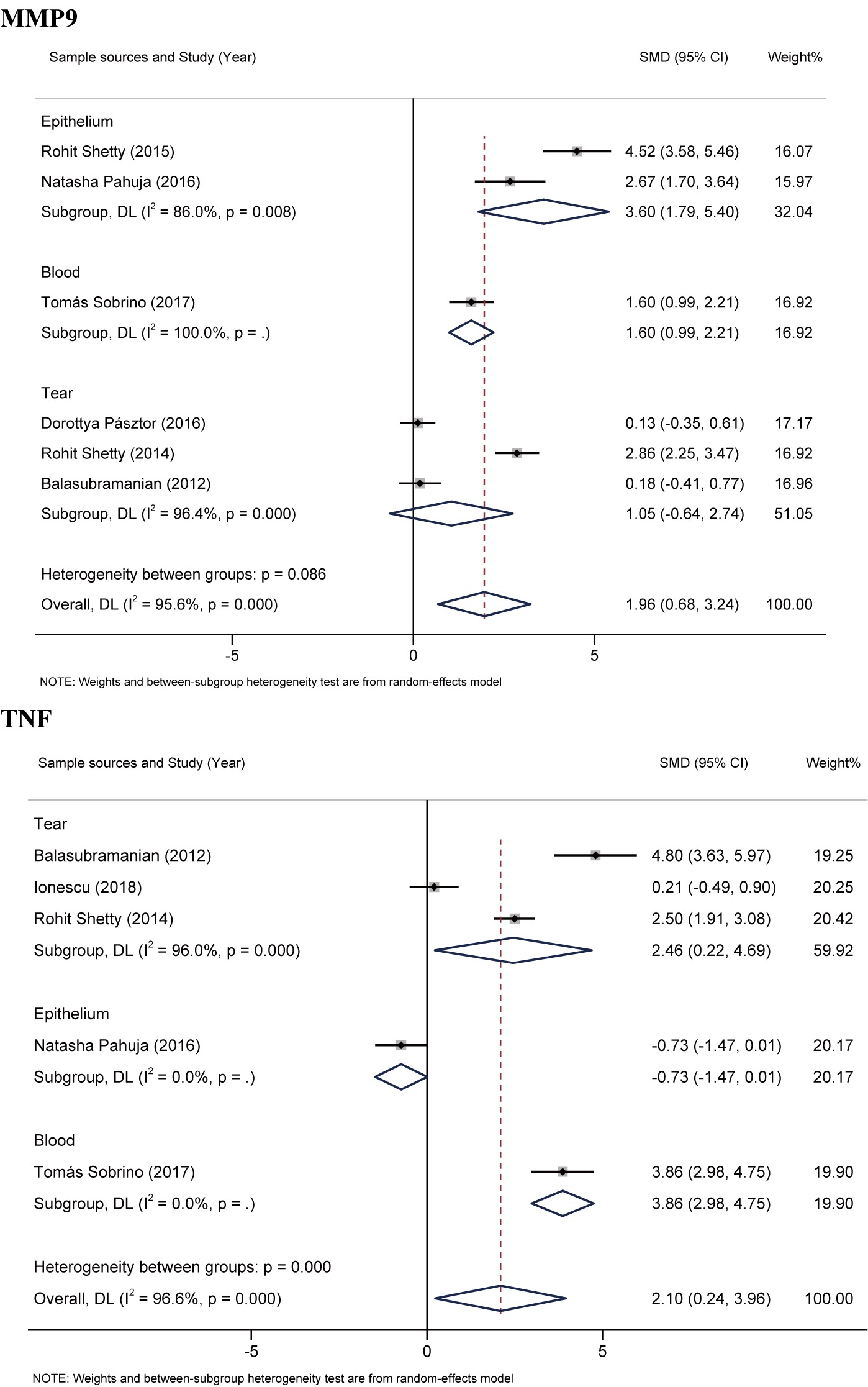

Supplement: S2 Fig — (TIF) [file pone.0299739.s003.tif]

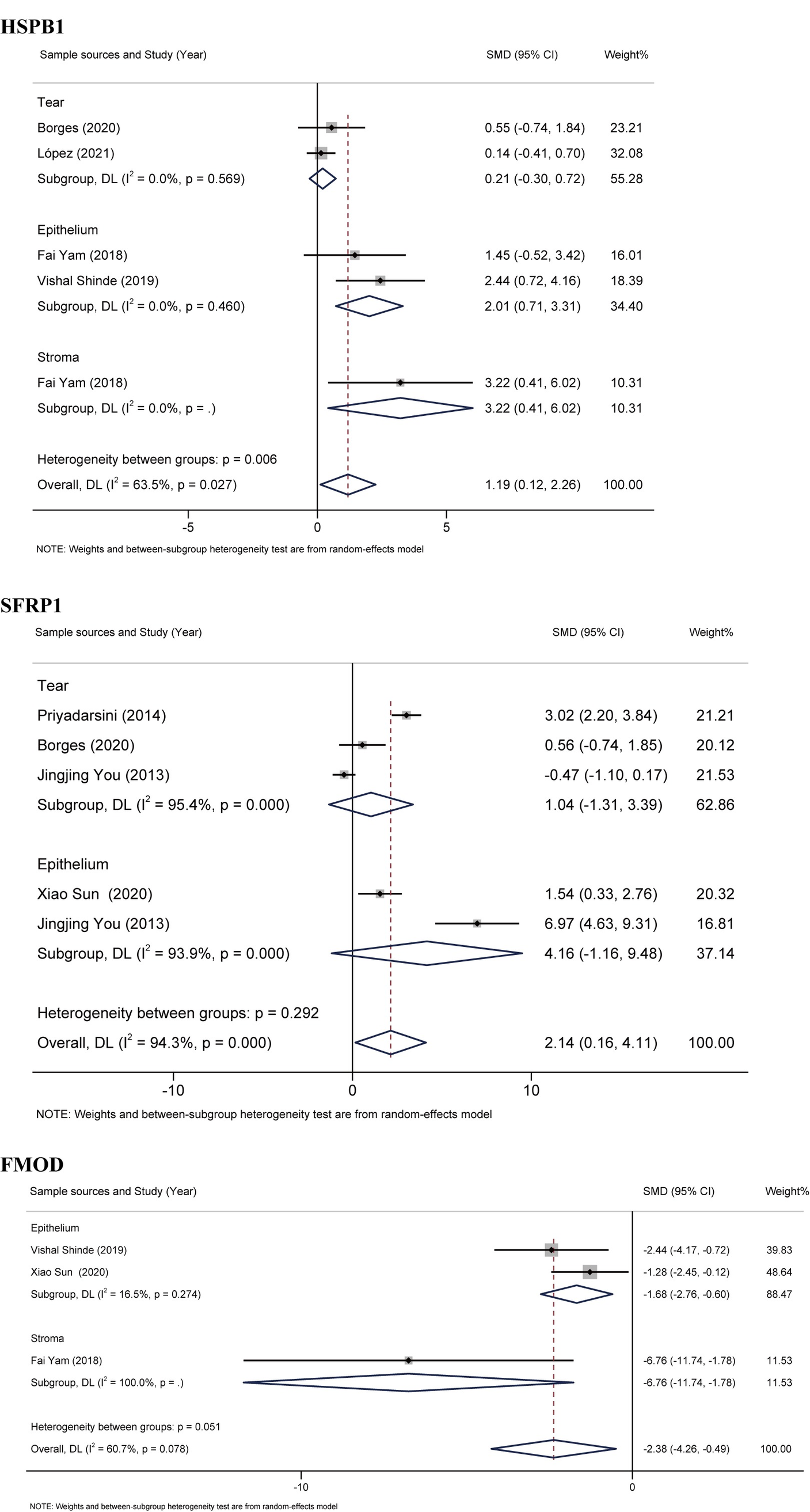

Supplement: S3 Fig — (TIF) [file pone.0299739.s004.tif]

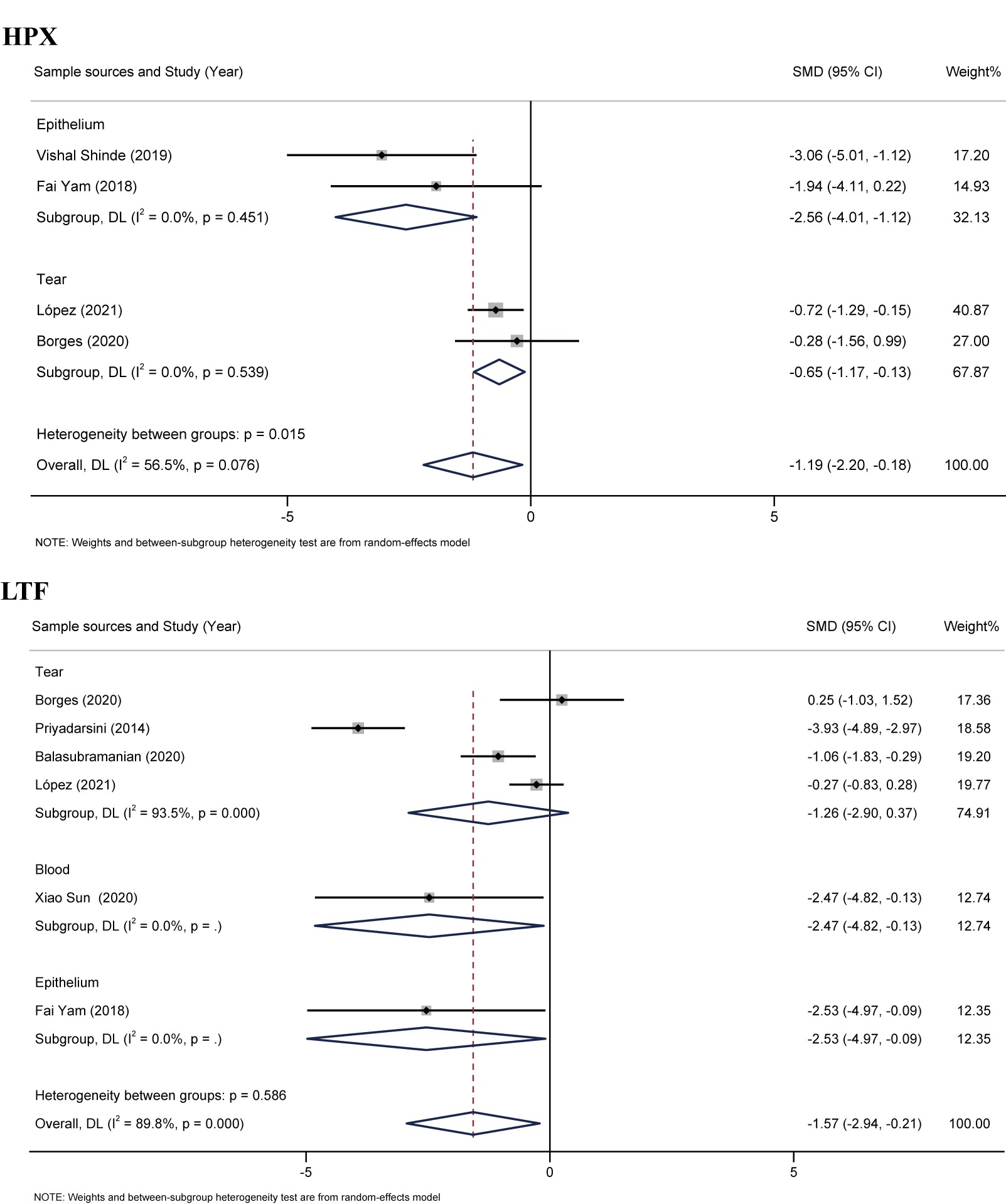

Supplement: S4 Fig — (TIF) [file pone.0299739.s005.tif]

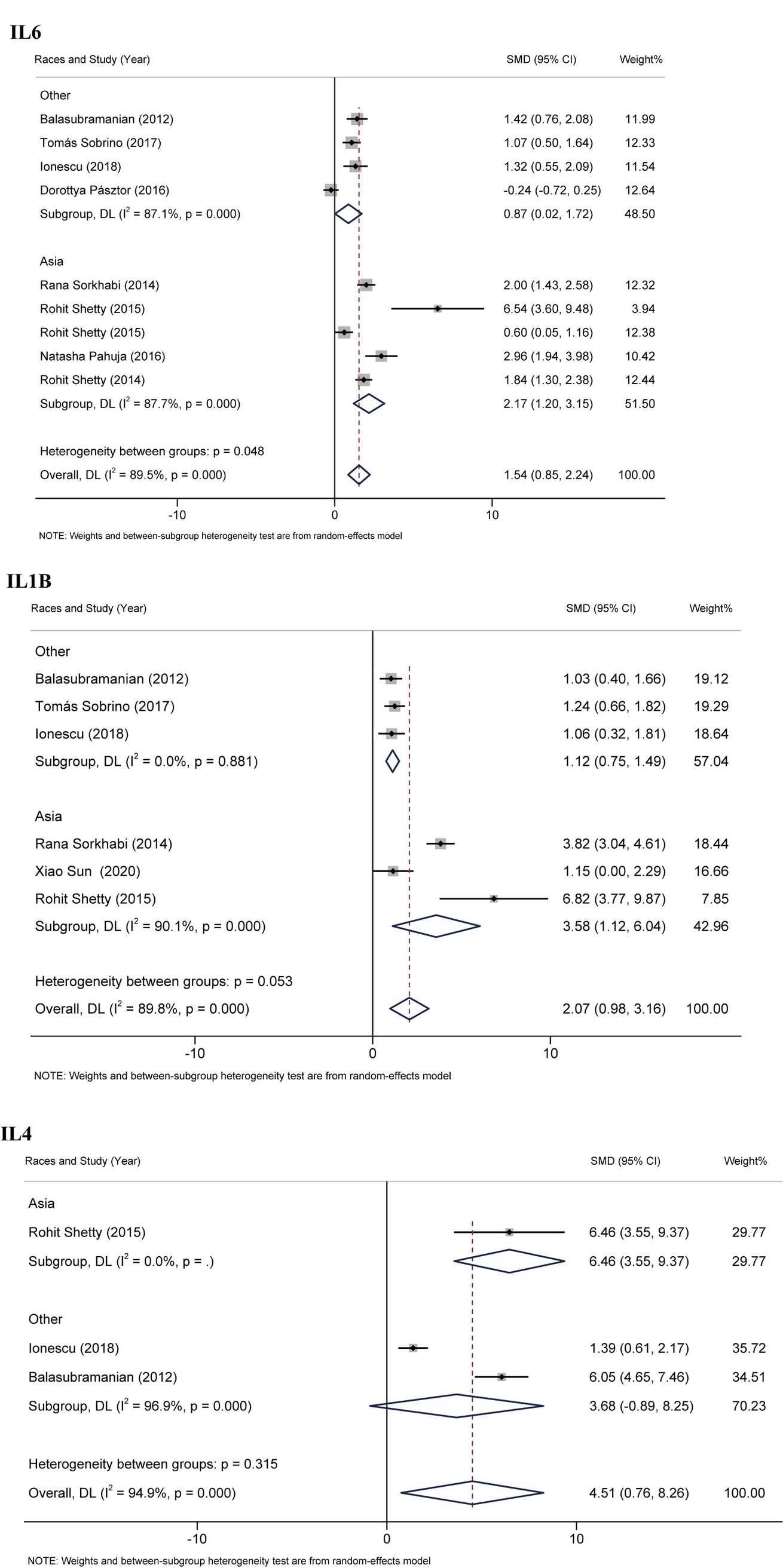

Supplement: S5 Fig — (TIF) [file pone.0299739.s006.tif]

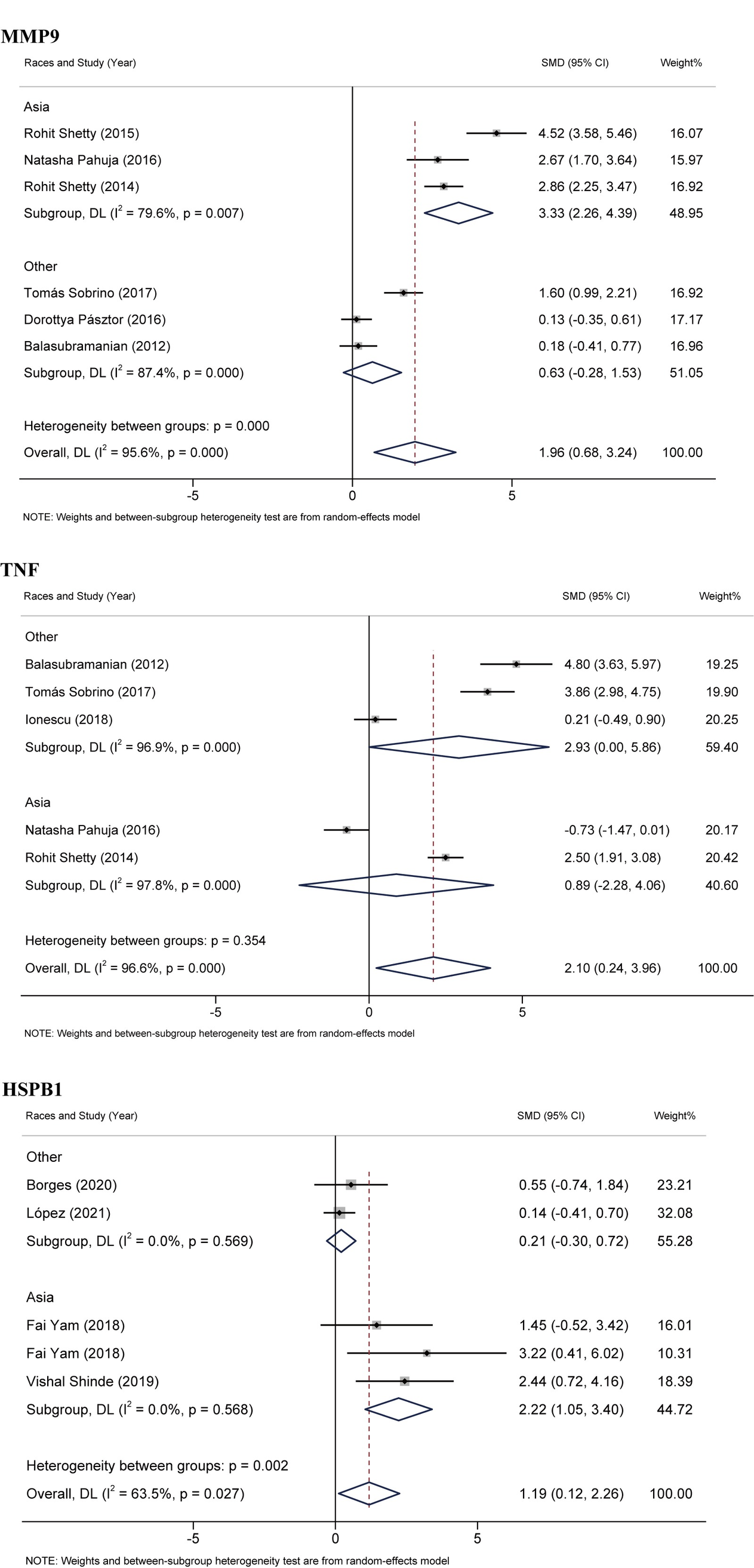

Supplement: S6 Fig — (TIF) [file pone.0299739.s007.tif]

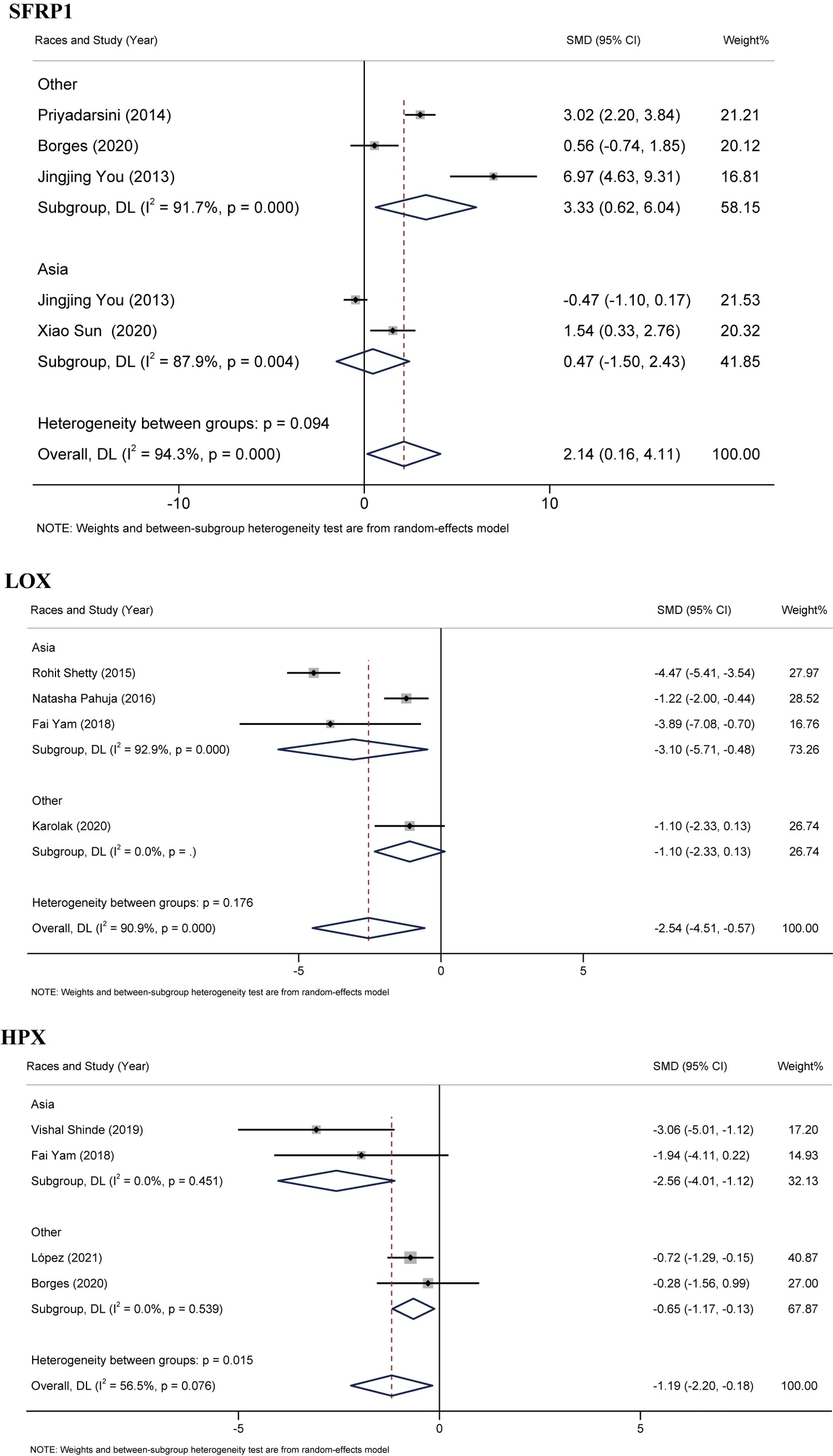

Supplement: S7 Fig — (TIF) [file pone.0299739.s008.tif]

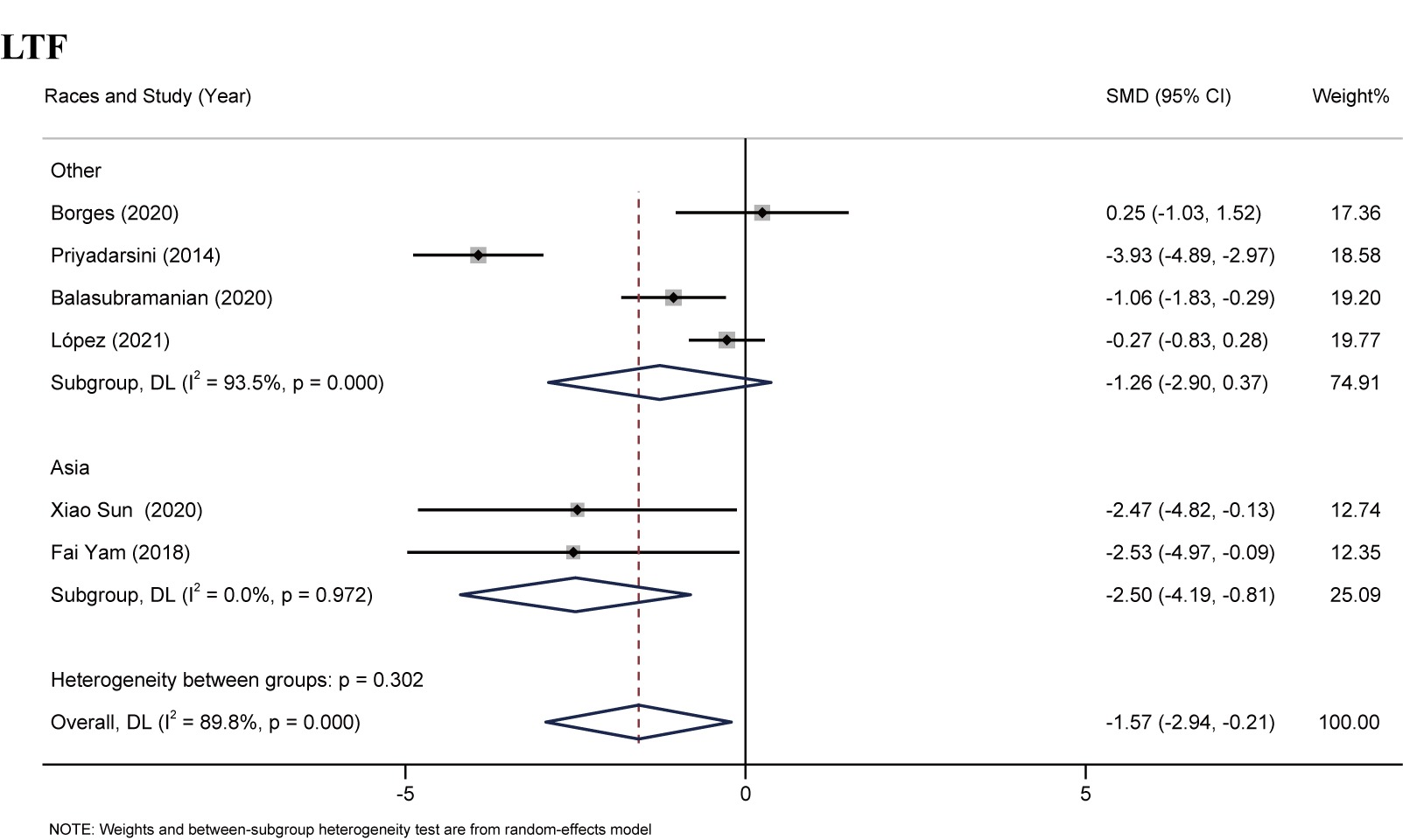

Supplement: S8 Fig — (TIF) [file pone.0299739.s009.tif]

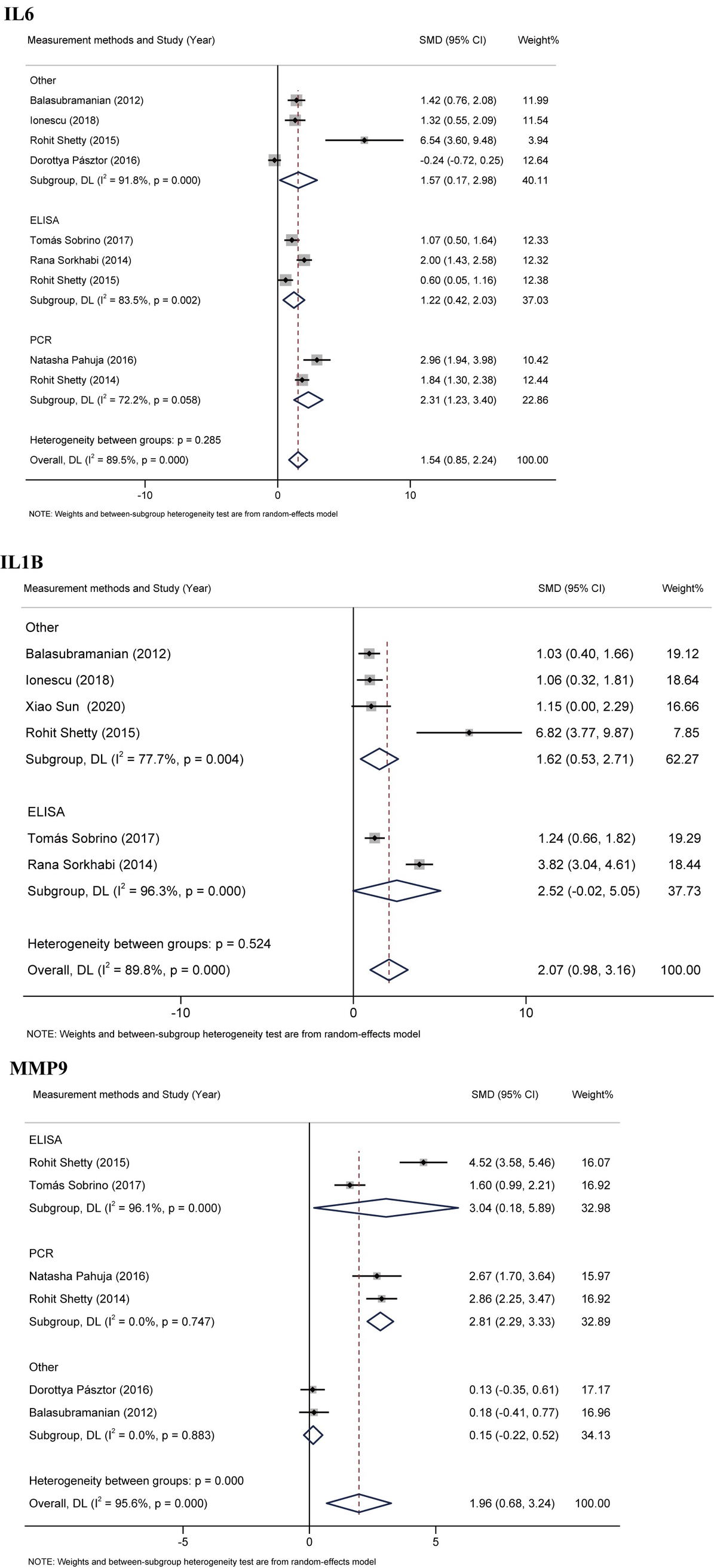

Supplement: S9 Fig — (TIF) [file pone.0299739.s010.tif]

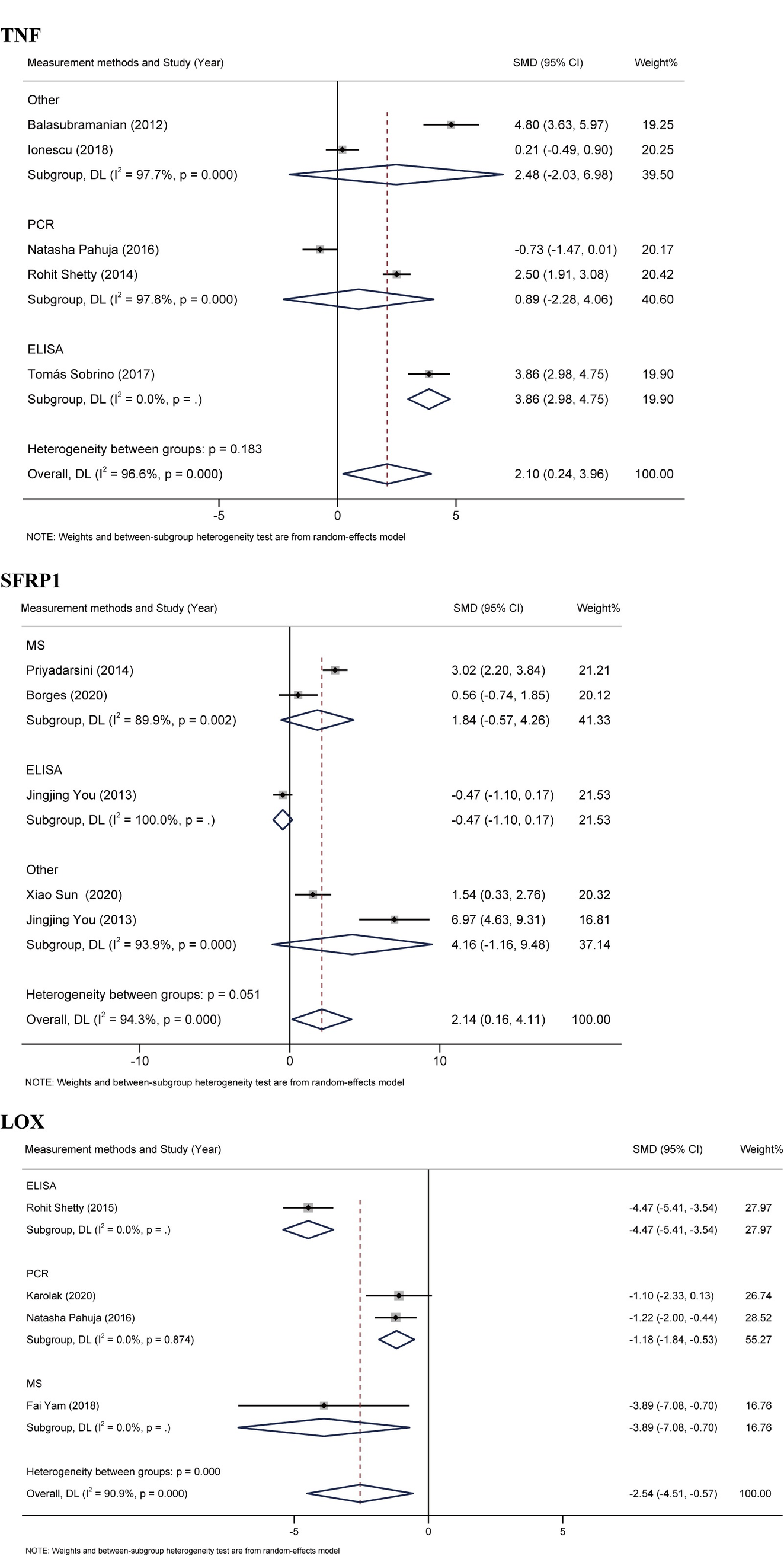

Supplement: S10 Fig — (TIF) [file pone.0299739.s011.tif]

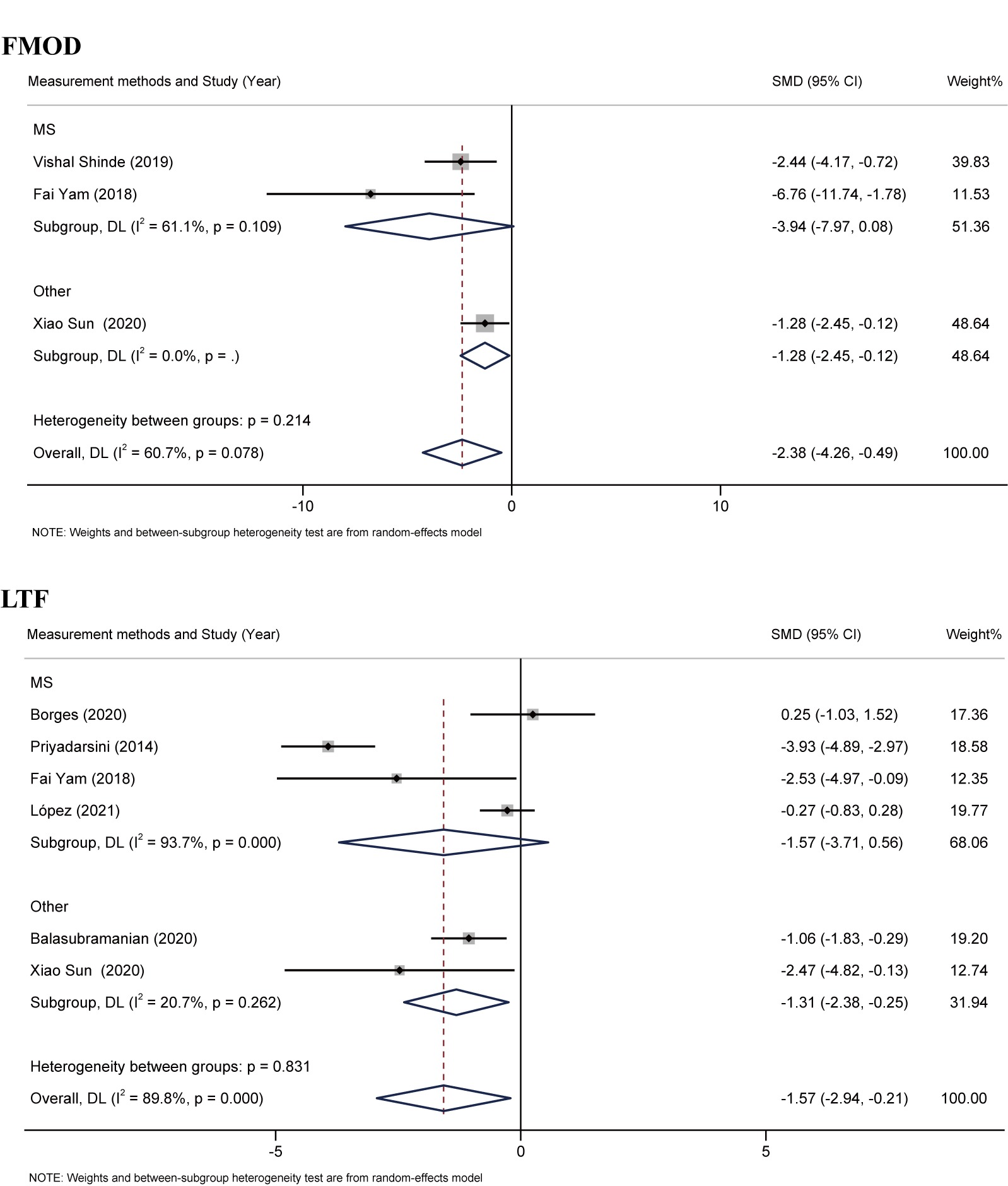

Supplement: S11 Fig — (TIF) [file pone.0299739.s012.tif]
