# Supplementary material for: The candidate proteins associated with keratoconus: A meta-analysis and bioinformatic analysis
Source: PLoS One. 2024 Mar 14;19(3):e0299739. doi: 10.1371/journal.pone.0299739 (PMC10939257; doi:10.1371/journal.pone.0299739)
Supplement: S1 File — (PDF) [file pone.0299739.s013.pdf]

Database	search formula	Items found
PubMed	(((("Keratoconus"[Mesh]) AND (((((((((((((((("Transcriptome"[Mesh]) OR (Transcriptomes[Title/Abstract])) OR (Gene Expression Profiles[Title/Abstract])) OR (Expression Profile, Gene[Title/Abstract])) OR (Expression Profiles, Gene[Title/Abstract])) OR (Gene Expression Profile[Title/Abstract])) OR (Profile, Gene Expression[Title/Abstract])) OR (Profiles, Gene Expression[Title/Abstract])) OR (Transcriptome Profiles[Title/Abstract])) OR (Profile, Transcriptome[Title/Abstract])) OR (Profiles, Transcriptome[Title/Abstract])) OR (Transcriptome Profile[Title/Abstract])) OR (Gene Expression Signatures[Title/Abstract])) OR (Expression Signature, Gene[Title/Abstract])) OR (Expression Signatures, Gene[Title/Abstract])) OR (Gene Expression Signature[Title/Abstract])) OR (Signature, Gene Expression[Title/Abstract])) OR (Signatures, Gene Expression[Title/Abstract]))) OR (("Keratoconus"[Mesh]) AND (("Proteome"[Mesh]) OR (Proteomes[Title/Abstract])))) OR (("Keratoconus"[Mesh]) AND (((((("Proteins"[Mesh]) OR (Protein[Title/Abstract])) OR (Gene Products, Protein[Title/Abstract])) OR (Protein Gene Products[Title/Abstract])) OR (Gene Proteins[Title/Abstract])) OR (Proteins, Gene[Title/Abstract])))) OR (("Keratoconus"[Mesh]) AND (((((((("Genes"[Mesh]) OR (Gene[Title/Abstract])) OR (Cistron[Title/Abstract])) OR (Cistrons[Title/Abstract])) OR (Genetic Materials[Title/Abstract])) OR (Genetic Material[Title/Abstract])) OR (Material, Genetic[Title/Abstract])) OR (Materials, Genetic[Title/Abstract]))) AND (2012:2023[pdat])	1267
Web of science	((TS=(Keratoconus)) AND (TS=(Transcriptome) OR AB=(Transcriptome OR Transcriptomes OR Gene Expression Profiles OR Expression Profile, Gene OR Expression Profiles, Gene OR Gene Expression Profile OR Profile, Gene Expression OR Profiles, Gene Expression OR Transcriptome Profiles OR Profile, Transcriptome OR Profiles, Transcriptome OR Transcriptome Profile OR Gene Expression Signatures OR Expression Signature, Gene OR Expression Signatures, Gene OR Gene Expression Signature OR Signature, Gene Expression OR Signatures, Gene Expression)) OR (TS=(Keratoconus)) AND (TS=(Proteome) OR AB=(Proteome OR Proteomes)) OR (TS=(Keratoconus)) AND (TS=(Proteins) OR AB=(Proteins OR Protein OR Gene Products, Protein OR Protein Gene Products OR Gene Proteins OR Proteins, Gene)) OR (TS=(Keratoconus)) AND (TS=(Genes) OR AB=(Genes OR Gene OR Cistron OR Cistrons OR Genetic Materials OR Genetic Material OR Material, Genetic OR Materials, Genetic))) AND (PY=(2023 OR 2022 OR 2021 OR 2020 OR 2019 OR 2018 OR 2017 OR 2016 OR 2015 OR 2014 OR 2013 OR 2012))	1169

**Cochrane**	#1 MeSH descriptor: [Keratoconus] explode all trees 349<br>#2 MeSH descriptor: [Transcriptome] explode all trees 377<br>#3 (Transcriptome or Transcriptomes or Gene Expression Profiles or Expression Profile, Gene or Expression Profiles, Gene or Gene Expression Profile or Profile, Gene Expression or Profiles, Gene Expression or Transcriptome Profiles or Profile, Transcriptome or Profiles, Transcriptome or Transcriptome Profile or Gene Expression Signatures or Expression Signature, Gene or Expression Signatures, Gene or Gene Expression Signature or Signature, Gene Expression or Signatures, Gene Expression):ti,ab,kw (Word variations have been searched) 6994<br>#4 #2 or #3 6994<br>#5 MeSH descriptor: [Proteomics] explode all trees 305<br>#6 (Proteome or Proteomes):ti,ab,kw (Word variations have been searched) 1594<br>#7 #5 or #6 1595<br>#8 MeSH descriptor: [Proteins] explode all trees 149931<br>#9 (Protein or Gene Products, Protein or Protein Gene Products or Gene Proteins or Proteins, Gene):ti,ab,kw (Word variations have been searched) 115102<br>#10 #8 or #9 219353<br>#11 MeSH descriptor: [Genes] explode all trees 2278<br>#12 (Genes or Gene or Cistron or Cistrons or Genetic Materials or Genetic Material or Material, Genetic or Materials, Genetic):ti,ab,kw (Word variations have been searched) 51240<br>#13 #11 or #12 51706<br>#14 #1 and #4 0<br>#15 #1 and #7 0<br>#16 #1 and #10 102<br>#17 #1 and #13 2<br>#18 #14 or #15 or #16 or #17 with Cochrane Library publication date Between Jan 2012 and Jan 2023, in Trials 88	88
**Embase**	#11. (#3 OR #5 OR #7 OR #9) AND [01-01-2012]/sd NOT [31-01-2023]/sd 515<br>#10. #3 OR #5 OR #7 OR #9 752<br>#9. #1 AND #8 541<br>#8. genes:ab,ti OR gene:ab,ti OR cistron:ab,ti OR cistrons:ab,ti OR 'genetic materials':ab,ti OR 'genetic material':ab,ti OR 'material, genetic':ab,ti OR 'materials, genetic':ab,ti 3,141,029<br>#7. #1 AND #6 388<br>#6. proteins:ab,ti OR protein:ab,ti OR 'gene 4,162,351	515

	26<br>products, protein':ab,ti OR 'protein gene products':ab,ti OR 'gene proteins':ab,ti OR 'proteins, gene':ab,ti<br>#5. #1 AND #4 20<br>#4. proteome:ab,ti OR proteomes:ab,ti 60,715<br>#3. #1 AND #2 18<br>#2. transcriptome:ab,ti OR transcriptomes:ab,ti OR 'gene expression profiles':ab,ti OR 'expression profile, gene':ab,ti OR 'expression profiles, gene':ab,ti OR 'gene expression profile':ab,ti OR 'profile, gene expression':ab,ti OR 'profiles, gene expression':ab,ti OR 'transcriptome profiles':ab,ti OR 'profile, transcriptome':ab,ti OR 'profiles, transcriptome':ab,ti OR 'transcriptome profile':ab,ti OR 'gene expression signatures':ab,ti OR 'expression signature, gene':ab,ti OR 'expression signatures, gene':ab,ti OR 'gene expression signature':ab,ti OR 'signature, gene expression':ab,ti OR 'signatures, gene expression':ab,ti 163,463<br>#1. keratoconus:ab,ti 9,472	
