# Supplementary material for: The candidate proteins associated with keratoconus: A meta-analysis and bioinformatic analysis
Source: PLoS One. 2024 Mar 14;19(3):e0299739. doi: 10.1371/journal.pone.0299739 (PMC10939257; doi:10.1371/journal.pone.0299739)
Supplement: S2 File — (PDF) [file pone.0299739.s014.pdf]

Genes	Sequences
IL6	F 5'- CCTGAACCTTCCAAAGATGGC-3'
	R 5'-CTTGGGGTTCTTGCTGATGT-3'
IL1B	F 5'-CAACAGGCTGCTCTGGGATTC-3'
	R 5'-AGCCATCATTTCACTGGCGA-3'
MMP9	F 5'-TCTACACCCAGGACGGCAAT-3'
	R 5'-GAAGCCGAAGAGCTTGTCCC-3'
TNF	F 5'-ACTTTGGAGTGATCGGCCC-3'
	F 5'-GCTTGAGGGTTTGCTACAACA-3'
LOX	F5'-CCTACTACATCCAGGCGTCCA-3'
	R5'-CATAATCTCTGACATCTGCCCTGT-3'
GAPDH	F 5'- ATTCCATGGCACCGTCAAGG-3'
	R 5'-CAGCATCGCCCCACTTGATT-3
