# Supplementary material for: The candidate proteins associated with keratoconus: A meta-analysis and bioinformatic analysis
Source: PLoS One. 2024 Mar 14;19(3):e0299739. doi: 10.1371/journal.pone.0299739 (PMC10939257; doi:10.1371/journal.pone.0299739)
Supplement: S3 File — (PDF) [file pone.0299739.s015.pdf]

Study(year)	Country	Design	Age (KC vs Control)	Male/Female ratio KCvs.Contro	Sample Size (KC vs Control)	Sample source	Methods of analysis	Protein	Source of the control	Determining the disease group
**López(2021)**	**Spain(Europe)**	cross-sectional study	44.88 (5.01) vs. 43.96 (6.94)	**60% vs. 60%**	**25 vs. 25**	**Tear**	**LC-MS/MS**	LTF, KRT1, AZGP1, LYZ, GSTP1, PRDX1, HSPB1, APOD, PGK1, PPIA, ANXA5, PIP, S100A9, MSLN, DEFA3, LCN2, ANXA2, IGKC, LDHA, CAP1, APOA1, IGHG2, , LCN1, IGHG1, IGHG3, ANXA1, ALDOA, HP, KRT9, SERPINA3, LGALS3BP, TTR, HPX, SERPINC1, C3, EZR, KRT19, ENO1, AKR1A1, CTSD, SCGB2A1, S100A11, PFN1, IGLL5, A1BG, PGM1, PSME1, PRDX2, FABP5, CNDP2, CLIC1,	Clinical parameters were normal, with no altered or irregular astigmatism assessed by tomography, and any control participants with a family history of KC were also excluded	Topography, chromatic aberration and tomography
**Karolak(2020)**	**Poland(Europe)**	NS	39.83(8.93) vs. 59.17(15.69)	**66% vs. 33%**	**6 vs. 6**	**Corneas**	RT-qPCR	LOX	Patients referred for corneal transplantation for different reasons	Complete ophthalmic evaluation
**Borges(2020)**	**Brazil(South America)**	NS	30.5 vs. 47.5	**50% vs. 16.67%**	**4 vs. 6**	**Tear**	**LC-MS/MS**	TIMP, LTF, KRT1, SFRP1, PIP, ORM2, ALB, AZGP1, LYZ, GSTP1, PRDX1, HSPB1, APOD, PGK1, PPIA, ANXA5, S100A9, MSLN, DEFA3, LCN2, PTGDS, CD9, GANAB, PGAM1, MYL6, MIF, ANXA2, IGKC, LDHA, CAP1, IGHA1, APOA1, IGHG2, LCN1, IGHG1, IGHG3, ANXA1, ALDOA, HP, KRT9, SERPINA3, LGALS3BP, TTR, HPX, SERPINC1, C3, EZR, KRT19, ENO1, AKR1A1, CTSD, SCGB2A1, S100A11, PFN1, IGLL5, A1BG, LYPD2, NUCB2, APOA4, APOH, ITIH2, ITIH1, IGHA2, PLTP, IGHM	Corneal tomograms and indices within normal limits, ocular surface parameters within normal limits, no clinical signs of pterygium or any other ocular surface disease	Imaging evaluation shows characteristic corneal thinning, thinning, corneal topographic changes and irregular astigmatism
**Xiao Sun (2020)**	**China(Asia)**	NS	16(2.45) vs. 20.14(1.95)	**57.14% vs. 57.14%**	**7 vs. 7**	**Epithelium tissue**	**RNA-Seq**	**TIMP1, KRT1, SFRP1, LYZ, CRIP1,** IL1b, SUSD2, S100A9, GSTM3, FMOD, XRCC5, GPX1, ARL8B, CKAP4, CNPY2, WDR1, CALR, DSG1, IDH3A, DPP3, RACK1, TBCA, PA2G4, GSTO1, TKT, PARK7, TGM2, PRDX5	People with mild myopia	Patients diagnosed with KC and undergoing de-epithelialized collagen cross-linking

**Xiao Sun (2020)**	**China(Asia)**	NS	14.67(3.06) vs. 20(1.73)	**33.33% vs. 100%**	**3 vs. 3**	**Blood**	**RNA-Seq**	**LTF, KRT1, SUSD2, MSLN, DEFA3, LCN2, PTGDX, CRIP1, ORM2,** FKBP2, CCS, ARPC4, ETHE1, FAU, NHLRC2, LGALS1, TUBB4B, HSPA1B, RCN3, TWF2, ENPP1	People with mild myopia	Patients diagnosed with KC and undergoing de-epithelialized collagen cross-linking
**Burcel(2020)**	**Romania(Europe)**	case–control study	26.13(8.79) vs. 26.57(9.79)	**68.75% vs. 64.29%**	**16 vs. 14**	**Tear**	**ELISA**	**ALB**	No change in corneal topography	Patients with KC diagnosed by biomicroscopy, keratometry, refractive error, and Amsler Krumreich classification of central corneal thickness
**Vishal Shinde(2019)**	**Saudi Arabia (Asia)**	case–control study	27.6(5.35) vs. 62.4(5.54)	**60% vs. 60%**	**5 vs. 5**	**Corneas**	**LC-MS/MS**	**TIMP, KRT1, AZGP1, LYZ, GSTP1, HBB, APOA1, PRDX1, HSPB1, APOD, PGK1, PRDX1, PPIA, ANXA5, IL1b, SUSD2, S100A9, LCN2, PTGDS, CD9, GANAB, PGAM1, MYL6, MIF, HP, KRT9, SERPINA3, LGALS3BP, TTR, HPX, SERPINC1, C3, EZR, KRT19, ENO1, AKR1A1, CTSD, SCGB2A1, S100A11, PFN1, IGLL5, A1BG, PGM1, PSME1, PRDX2, FABP5, CNDP2, CLIC1, TIMP2, CRIP1, GSTM3, FMOD, ORM2, FAU, NHLRC2, LGALS1, RCN3, TWF2, ENPP1, APOA4, APOH, ITIH2, ITIH1, ALDOC, TPM3, TSN, HDHD2, C1QBP, RPN1, LYPD3, VAT1, LMNB1, NDRG1, PURA, ADIRF, RAB10, PEBP1, LAMP1, ARF4, XRCC6, RAB14, MRC2, PPIB, KERA, ERP29, NIT2, LMNA, DCTN2, GPC1, CBR1, ACTN4, TLN1, PDIA3, LSM3, PCBP1, LUM, NAMPT, PYGB, MTAP, OUTB1, MESD, XRCC5, GPX1, ARL8B, CKAP4, CNPY2, WDR1,**	Corneas unsuitable for transplantation	Obtained after penetrating corneal transplantation or lamellar corneal transplantation, cone cornea diagnosis is performed by trained corneal specialists from KKESH.

								**CALR, DSG1, IDH3A, DPP3, RACK1, TBCA, PA2G4, GSTO1**		
**Fai Yam(2018)**	**Singapore(Asia)**	NS	25.3(5.1) vs. 30(4)	**NS**	**4 vs. 2**	**Epithelium tissue**	**SWATH-MS**	**LTF, KRT1, AZGP1, LYZ, GSTP1, PRDX1, APOD, PTGDS, CD9, GANAB, PGAM1, MYL6, MIF, ANXA2, IGKC, LDHA, CAP1, IGHA1, APOA1, IGHG2, LCN1, IGHG1, IGHG3, ANXA1, ALDOA, HP, KRT9, SERPINA3, LGALS3BP, TTR, HPX, SERPINC1, C3, EZR, KRT19, ENO1, AKR1A1, CTSD, SCGB2A1, S100A11, PFN1, IGLL5, A1BG, PGM1, PSME1, PRDX2, FABP5, CNDP2, CLIC1, ALB, PGK1, PRDX1, PPIA, ANXA5, LOX, MSLN, DEFA3, LYPD2, NUCB2, ALDOC, TPM3, TSN, HDHD2, C1QBP, RPN1, LYPD3, VAT1, LMNB1, NDRG1, PURA, ADIRF, RAB10, PEBP1, LAMP1, ARF4, XRCC6, RAB14, MRC2, PPIB, KERA, ERP29, NIT2, LMNA, DCTN2, GPC1, CBR1, ACTN4, TLN1, PDIA3, LSM3, PCBP1, LUM, NAMPT, PYGB, MTAP, OUTB1, MESD, XRCC5, GPX1, ARL8B, CKAP4, CNPY2, WDR1, CALR, DSG1, IDH3A, DPP3, RACK1, TBCA, PA2G4, GSTO1, TKT, PARK7, TGM2, PRDX5, FAU, NHLRC2, LGALS1, TUBB4B, HSPA1B**	Normal corneal epithelium	Assessment of slit lamp biomicroscopy, corneal topography and clinical signs
**Fai Yam(2018)**	**Singapore(Asia)**	NS	25.3(5.1) vs. 34(3)	**NS**	**4 vs. 2**	**Stroma tissue**	**SWATH-MS**	**TIMP, GSTP1, PRDX1, APOD, SUSD2, PTGDS, CD9,** GANAB, PGAM1, MYL6, MIF, ANXA2, IGKC, LDHA, CAP1, IGHA1, APOA1, IGHG2, LCN1, IGHG1, IGHG3, ANXA1, ALDOA, HP, KRT9, SERPINA3, LGALS3BP, TTR, HPX, SERPINC1, C3, EZR, KRT19, ENO1, AKR1A1, CTSD, SCGB2A1, S100A11, PFN1, IGLL5, A1BG, PGM1, PSME1, PRDX2, FABP5, CNDP2, CLIC1, TIMP2, GSTM3, FMOD, ORM2, FKBP2, CCS, ARPC4, ETHE1, ALB, RCN3, TWF2, ENPP1, NUCB2, APOH, ITIH2, ITIH1,	Normal corneal stroma	Assessment of slit lamp biomicroscopy, corneal topography and clinical signs

								IGHA2, PLTP, IGHM, ALDOC, TPM3, TSN, HDHD2, C1QBP, RPN1, LYPD3, VAT1, LMNB1, NDRG1, PURA, ADIRF, RAB10, PEBP1, LAMP1, ARF4, XRCC6, RAB14, MRC2, PPIB, KERA, ERP29, NIT2, LMNA, DCTN2, GPC1, CBR1, ACTN4, TLN1, PDIA3, LSM3, PCBP1, LUM, NAMPT, PYGB, MTAP, OUTB1, MESD, XRCC5, GPX1, ARL8B, CKAP4, CNPY2, WDR1, CALR, DSG1, IDH3A, DPP3, RACK1, TBCA, PA2G4, GSTO1, TKT, PARK7, TGM2, PRDX5, APOA2, HBB		
**Ionescu(2018)**	**Romania(Europe)**	cross-sectional study	23.35(11.80) vs. 28.66(3.03)	**64.71% vs. 40%**	**17 vs. 15**	**Tear**	**MILLIPLEX MAP**	**IL6, IL10, TNF, IL1B, IL4**	Normal biomicroscopy, normal corneal topography readings and normal biomechanical measurements	Corneal Topography, Thickness Measurements, Corneal Biomechanics, and Slit Lamp Examination to Evaluate a Positive Diagnosis of Conical Cornea
**Tomás Sobrino(2017)**	**Chile(South America)**	cross-sectional study	33.1(10.9) vs. 30.4(7.6)	**55% vs. 55%**	**40 vs. 20**	**Blood**	**ELISA**	**IL1B, IL6, TNF, MMP9**	Normal corneal topography	Biomicroscopic signs in both eyes, such as Vogt's lines, Fleischer's rings, prominent corneal nerves
**Natasha Pahuja(2016)**	**India(Asia)**	NS	NS	**NS**	**66 vs. 23**	**Epithelium cell**	**PCR**	**LOX, MMP9, TIMP1, IL6, IL10**	Corneas undergoing refractive keratectomy (PRK)	Diagnosis of KC by retinoscopy, slit lamp biomicroscopy and keratometry
**Dorottya**	**Hungary(Eur**	cross-sectio	44.2 vs. 44.5	**NS**	**55 vs. 24**	**Tear**	**CBA**	**IL6,MMP9, TIMP1,IL10**	Received a complete ophthalmologic	Thinning of the central

**Pásztor(2016)**	**ope)**	nal study							evaluation	or paracentral interstitium of the cornea, conical prominence, Fleischer's rings, Vogt's lines on slit lamp examination, and topographic changes
**Rohit Shetty(2015)**	**India(Asia)**	NS	22.7(5.7) vs. 22.7(5.7)	**NS**	**12 vs. 10**	Epithelial cells	**PCR**	**MMP9, IL6, TNF**	Subjects without clinical signs of KC treated with PRK	NS
**Rohit Shetty(2015)**	**India(Asia)**	NS	28.8 (2) vs. 28.0 (3)	**27.27% vs. 33.33%**	**7 vs. 6**	**Tear**	**CBA**	**IL6, IL10, IL1B, IL4**	NS	KC diagnosis using retinoscopy, keratoconus and slit lamp biomicroscopy
**Priyadarsini(2014)**	**Denmark(Europe)**	NS	30(10.58) vs. 33(8.58)	**NS**	**17 vs. 36**	**Tear**	**LC-MS/MS**	**LTF, SFRP1, AZGP1, PIP**	NS	Refraction, best-corrected visual acuity measurement, slit lamp examination and pentacam HR puncture tomography
**Sorkhabi(2014)**	**Iran(Asia)**	case–control study	24.09(6.50) vs. 24.43(4.55)	**57.14% vs. 43.33%**	**42 vs. 30**	**Tear**	**ELISA**	**IL6, IL1B, IL10**	NS	Clinical examination and corneal topography to determine the presence of KC
**Rohit Shetty(2014)**	**India(Asia)**	NS	24.6(8.4) vs. 24.6(8.4)	**NS**	**90 vs. 52**	**Epithelium cell**	**ELISA**	**LOX, MMP9, LOX**	Subjects with normal corneal topography	Epithelial cells collected after corneal collagen cross-linking or T-PRK

										in KC patients
**Jingjing You(2013)**	**Australia(Oceania)**	NS	21(5.8) vs. 53(8.3)	**46.67% vs. 57.14%**	**15 vs. 7**	**Epithelium cell**	**IHC**	**SFRP1**	NS	KC patients were previously diagnosed on the basis of clinical signs and corneal topography.
**Jingjing You(2013)**	**China(Asia)**	NS	25.8(6.7) vs. 30.9(7.5)	**57.58% vs. 46.88%**	**33 vs. 33**	**Tear**	**ELISA**	**SFRP1**	NS	The diagnosis was made on the basis of clinical signs and corneal topography
**Balasubramanian(2012)**	**Australia(Oceania)**	NS	27.4(6.0) vs. 29.8(8.9)	**NS**	**25 vs. 20**	**Tear**	**Anti body Chip**	**MMP9, TIMP1, IL6, IL10, LTF, TNF, IL1B,IL4,TIMP2**	NS	NS
