# Supplementary material for: The candidate proteins associated with keratoconus: A meta-analysis and bioinformatic analysis
Source: PLoS One. 2024 Mar 14;19(3):e0299739. doi: 10.1371/journal.pone.0299739 (PMC10939257; doi:10.1371/journal.pone.0299739)
Supplement: S4 File — (PDF) [file pone.0299739.s016.pdf]

	Study	Year		KC			NC		
				n1	mean1	sd1	n2	mean2	sd2
IL6	Balasubramanian	2012	translational	25	313.6	232.2	20	66.7	8.7
	Tomás Sobrino	2017	translational	40	112.4	29.6	20	75.9	41.6
	Rana Sorkhabi	2014	translational	42	17.49	1.92	30	13.81	1.71
	Ionescu	2018	translational	17	122.32	18.63	15	100.31	14.13
	Rohit Shetty	2015	translational	7	2413	489	6	49	21
	Rohit Shetty	2015	translational	17	7.405	1.482	52	6.488	1.534
	Natasha Pahuja	2016	transcription	11	6.84	3.67	23	0.52	0.71
	Dorottya Pásztor	2016	translational	55	160.2	265.7	24	217.1	172.2
	Rohit Shetty	2014	transcription	94	4.05	0.73	20	2.65	0.89
IL1b	Balasubramanian	2012	translational	25	67.4	14.2	20	51.7	16.4
	Tomás Sobrino	2017	translational	40	123.9	53.8	20	63	38.1
	Rana Sorkhabi	2014	translational	42	8.58	1.15	30	4.98	0.52
	Ionescu	2018	translational	17	113.52	34.86	15	83.45	18.07
	Xiao Sun	2020	transcription	7	5.516502085	6.105589959	7	0.534123376	0.68156749
	Rohit Shetty	2015	translational	7	2474	486	6	24	21
IL4	Rohit Shetty	2015	translational	7	3932	819	6	23	17
	Ionescu	2018	translational	17	461.67	283.2	15	159.21	99.72
	Balasubramanian	2012	translational	25	72.4	4.5	20	46.7	3.9
MMP9	Rohit Shetty	2015	translational	17	6.246	1.697	52	1.773	0.621
	Natasha Pahuja	2016	transcription	11	0.33	0.09	23	0.18	0.03

	Tomás Sobrino	2017	translational	40	25.9	6.4	20	16.8	3.8
	Dorottya Pásztor	2016	translational	55	51.3	131.9	24	36.7	61.5
	Rohit Shetty	2014	transcription	94	5.16	0.95	20	2.47	0.89
	Balasubramanian	2012	translational	25	442.1	474.5	20	349.1	545.3
TNFa	Balasubramanian	2012	translational	25	92.2	8.5	20	54.4	7.0
	Natasha Pahuja	2016	transcription	11	0.16	0.06	23	0.22	0.09
	Tomás Sobrino	2017	translational	40	93.2	5.9	20	35.9	24.5
	Ionescu	2018	translational	17	131.8	34.29	15	125.33	28.08
	Rohit Shetty	2014	transcription	94	1.88	0.59	20	0.53	0.14
HSPB1	Borges	2020	translational	4	24.76	2.43	6	20.28	10.12
	Fai Yam	2018	translational	4	58922317.45	19429644.07	2	34500866.99	437542.735
	Fai Yam	2018	translational	4	2791092.173	657276.3083	2	929288.5621	211408.95
	López	2021	translational	25	1083193.2	938382.3114	25	979461.6	437500.8481
	Vishal Shinde	2019	translational	5	122.86	25.20298395	5	77.14	8.219391705
SFRP1	Priyadarsini	2014	translational	17	2.87E+06	5.27E+05	36	1280000	526000
	Borges	2020	translational	4	24.08	1.18	6	19.77	9.78
	Jingjing You	2013	translational	19	3.41	3.12	20	5.55	5.62
	Xiao Sun	2020	transcription	7	196.9668766	171.9257761	7	8.949394188	13.41930889
	Jingjing You	2013	translational	15	4.2	0.4	7	1.6	0.3
LOX	Rohit Shetty	2015	translational	17	0.602	0.105	52	2.122	0.385
	Karolak	2020	transcription	6	0.36	0.18	6	1.38	1.3

	Natasha Pahuja	2016	transcription	11	0.66	0.19	23	0.98	0.29
	Fai Yam	2018	translational	4	6333626.207	4791932.372	2	31631098.36	10009465.35
FMOD	Vishal Shinde	2019	translational	5	68.52	9.556442853	5	131.48	35.16978248
	Fai Yam	2018	translational	4	3259228.924	1809124.435	2	14875270.12	1415951.345
	Xiao Sun	2020	transcription	7	1.197481118	1.544832018	7	8.465477172	7.869987606
HPX	Vishal Shinde	2019	translational	5	74.44	5.157751448	5	125.56	23.03081414
	López	2021	translational	25	37696.648	32731.80394	25	**72839.68**	**60602.90905**
	Fai Yam	2018	translational	4	20965.67269	10528.4415	2	276082.9109	261898.7762
	Borges	2020	translational	4	25.422055	2.820299141	6	26.06252	1.82522597
LTF	Borges	2020	translational	4	35.88	0.29	6	35.62	1.32
	Priyadarsini	2014	translational	17	21200	943	36	14000000000	4300000000
	Balasubramanian	2020	translational	16	0.2	0.21	14	1.06	1.17
	Xiao Sun	2020	transcription	3	186.9677082	65.77970172	3	1277.224288	620.0584637
	Fai Yam	2018	translational	4	251357.2922	126658.0411	2	19652833.83	15319480.36
	López	2021	translational	25	26948260	11085837.9	25	30218648	12653190.98
VAT1	Gary Hin-Fai Yam	2018	translational	4	871035.0092	652251.9111	2	2631827.855	380336.0915
	Gary Hin-Fai Yam	2018	translational	4	231745.4693	121809.7927	2	353591.0815	50962.4517
	Vishal Shinde	2019	translational	5	88.38	7.182868508	5	111.62	11.19471304
NDRG1	Gary Hin-Fai Yam	2018	translational	4	28160.95835	28477.42976	2	140324.9655	60454.50242
	Gary Hin-Fai Yam	2018	translational	4	42670.10052	8247.774485	2	63777.88813	16441.2002

	Vishal Shinde	2019	translational	5	92.28	5.530786563	5	107.72	10.55848474
FKBP2	Gary Hin-Fai Yam	2018	translational	4	50945.28598	42753.2881	2	130418.7532	104961.7109
	Gary Hin-Fai Yam	2018	translational	4	73488.13885	69142.54411	2	247739.3091	76336.0163
	Xiao Sun	2020	transcription	3	190.8460614	43.27014416	3	414.9388114	237.7848619
MRC2	Gary Hin-Fai Yam	2018	translational	4	58921.07898	34502.39539	2	135419.7816	40688.76232
	Gary Hin-Fai Yam	2018	translational	4	128425.8161	48757.86323	2	246909.1481	153571.8371
	Vishal Shinde	2019	translational	5	95.78	6.655944711	5	104.22	13.51730742
KERA	Gary Hin-Fai Yam	2018	translational	4	772652.0886	501387.7635	2	712999.7097	363967.2533
	Gary Hin-Fai Yam	2018	translational	4	710544088.6	114044736.7	2	1004843880	182443964.1
	Vishal Shinde	2019	translational	5	91.44	5.372746039	5	108.58	12.91067775
CNPY2	Xiao Sun	2018	translational	4	17261.67147	14815.53866	2	44719.31614	11267.38511
	Gary Hin-Fai Yam	2018	translational	4	29049.05848	28886.34213	2	98010.98294	22172.29397
	Vishal Shinde	2019	translational	5	96.42	17.13562371	5	103.6	16.55983092
LYPD3	Gary Hin-Fai Yam	2018	translational	4	385278.7393	322640.0424	2	1504671.475	95104.714
	Gary Hin-Fai Yam	2018	translational	4	357519.3032	226723.215	2	557198.4297	98701.3139
	Vishal Shinde	2019	translational	5	97.46	4.975781346	5	102.54	9.465009245
