# Supplementary material for: The candidate proteins associated with keratoconus: A meta-analysis and bioinformatic analysis
Source: PLoS One. 2024 Mar 14;19(3):e0299739. doi: 10.1371/journal.pone.0299739 (PMC10939257; doi:10.1371/journal.pone.0299739)
Supplement: S5 File — (PDF) [file pone.0299739.s017.pdf]

Genes	No.of study	Pooled sample size		Outcome		Heterogeneity	
		case	control	P	SMD	p	I^2(95%)
IL6	9	308	210	0.000	1.54(0.85，2.24)	0	89.50%
TIMP1	7	107	83		-0.19(-1.04,0.66)	0	81.00%
MMP9	7	248	179	0.000	1.30(1.05,1.55)	0	96.50%
IL10	6	157	118		0.92(-0.74,2.59)	0	96.50%
LTF	6	69	86	0.024	-1.57(-2.94，-0.21)	0	89.80%
KRT1	6	48	48		-0.40(-1.35,0.54)	0.005	0.702
SFRP1	5	62	76	0.034	2.14(0.16,4.11)	0	94.3
AZGP1	5	55	74	0.937	-0.06(-1.52,1.41)	0	0.896
LYZ	5	45	45	0.508	-0.25(-1.00,0.49)	0.079	0.522
GSTP1	5	42	40	0.276	0.74(-0.59,2.06)	0.006	0.723
PRDX1	5	42	40	0.551	0.44(-1.01,1.89)	0.02	0.77
HSPB1	5	42	40	0.03	1.19(0.12,2.26)	0.027	63.5
APOD	5	42	40	0.909	0.04(-0.69,0.78)	0.132	0.434
PGK1	5	42	40	0.685	0.21(-0.79,1.21)	0.037	0.609
PPIA	5	42	40	0.429	0.62(-0.92，2.16)	0.001	0.779
ANXA5	5	42	40	0.1	-0.77(-1.69,0.15)	0.066	0.547
LOX	4	38	83	0.011	-2.32(-2.85,-1.79)	0	0.909
TNFa	7	239	149	0.027	1.08(0.78,1.37)	0	96.5
PIP	4	51	72		-1.12(-3.09，0.84)		
IL1b	6	138	98		2.07(0.98,3.16)	0	89.8%%
SUSD2	4	19	17	0.703	0.24(-0.98,1.46)	0.059	0.597
S100A9	4	41	43	0.654	0.15(-0.51,0.82)	0.156	0.425
MSLN	4	34	36	0.671	-0.36(-2.05,1.32)	0.019	0.748
DEFA3	4	34	36	0.461	-0.45(-1.63,0.74)	0.036	0.65
LCN2	4	34	36		-0.01(-0.48，0.46)		
PTGDS	4	16	12		-0.64(-3.01,1.73)	0.006	0.756
CD9	4	17	15	0.212	-0.48(-1.22,0.27)	0.436	0
GANAB	4	17	15	0.859	-0.065(-0.785,0.655)	0.81	0
PGAM1	4	17	15	0.552	-0.434(-1.865,0.997)	0.048	0.622
MYL6	4	17	15	0.586	0.499(-1.297,2.295)	0.01	0.735
MIF	4	17	15	0.233	-0.759(-2.006,0.487)	0.086	0.545
ANXA2	4	37	35	0.398	-0.647(-2.147,0.853)	0.01	0.737
IGKC	4	37	35	0.908	0.028(-0.443,0.499)	0.676	0
LDHA	4	37	35	0.474	-0.514(-1.922,0.89	0.015	0.711

					4)		
CAP1	4	37	35	0.223	-0.738(-1.924,0.448)	0.042	0.633
IGHA1	4	37	35	0.841	-0.048(-0.520,0.423)	0.611	0
APOA1	4	37	35	1	0.000(-0.717,0.717)	0.239	0.289
IGHG2	4	37	35	0.288	-0.255(-0.726,0.216)	0.859	0
LCN1	4	37	35	0.676	0.223(-0.822,1.268)	0.077	0.562
IGHG1	4	37	35	0.102	-0.396(-0.870,0.079)	0.842	0
IGHG3	4	37	35	0.334	-0.308(-0.933,0.317)	0.306	0.171
ANXA1	4	37	35	0.605	-0.334(-1.599,0.932)	0.03	0.665
ALDOA	4	37	35	0.926	0.059(-1.169,1.286)	0.036	0.649
HP	4	38	38		-0.30(-0.76，0.17)	0.212	0.333
KRT9	4	41	43		-0.10(-0.56,0.35)		
SERPINA3	4	41	43		-0.66(-2.12,0.80)		
LGALS3BP	4	41	43		-0.41(-1.33,0.52)		
TTR	4	41	43		-0.41(-1.27,0.46)		
HPX	4	41	43	0.022	-1.19(-2.20,-0.18)	0.076	0.565
SERPINC1	4	41	43		-0.31(-0.78，0.17)		
C3	4	41	43	0.349	-0.40(-1.23,0.43)	0.121	0.484
EZR	4	41	43	0.489	0.68(-1,24,2.60)	0	0.843
KRT19	4	41	43		-0.16(-0.62,0.30)		
ENO1	4	41	43		0.32(-1.55,2.19)		
AKR1A1	4	41	43		0.18(-1.22,1.57)		
CTSD	4	41	43		0.22(-0.66,1.11)		
SCGB2A1	4	41	43		0.20(-1.41,1.82)		
PFN1	4	41	43		0.75(-1.09,2.58)		
IGLL5	4	38	38	0.553	-0.286(-1.231,0.659)	0.061	0.593
PGM1	4	38	38	0.177	-0.903(-2.213,0.407)	0.03	0.664
PSME1	4	38	38	0.858	0.141(-1.402,1.685)	0.01	0.738
PRDX2	4	38	38	0.841	0.149(-1.306,1.604)	0.009	0.739
FABP5	4	38	38	0.598	0.477(-1.295,2.250	0.004	0.774

					)		
CNDP2	4	38	38	0.789	-0.218(-1.815,1.378)	0.006	0.758
CLIC1	4	38	38	0.696	0.356(-1.429,2.142)	0.004	0.772
IL4	3	49	41	0.018	4.51(0.76,8.26)	0	94.9
TIMP2	3	34	27		-2.49(-6.91,1.92)	0	0.923
CRIP1	3	15	15	0.514	0.615(-1.231,2.461)	0.014	0.767
GSTM3	3	16	16	0.446	-0.859(-3.065,1.348)	0.003	0.824
FMOD	3	16	14		-2.38(-4.46,-0.49)	0.078	0.607
ORM2	3	12	14	0.052	-1.469(-2.952,0.014)	0.099	0.568
FKBP2	3	11	7	0.009	-1.55(-2.71,-0.39)	0.7	0
CCS	3	11	7	0.075	-1.774(-3.727,0.178)	0.118	0.531
ARPC4	3	11	7	0.316	-0.736(-2.176,0.704)	0.177	0.423
ETHE1	3	11	7	0.181	-1.849(-4.558,0.860)	0.026	0.726
FAU	3	12	10	0.887	-0.211(-3.128,2.706)	0.001	0.852
NHLRC2	3	12	10	0.997	0.006(-3.446,3.458)	0.004	0.82
LGALS1	3	12	10	0.123	-1.429(-3.246,0.388)	0.081	0.602
TUBB4B	3	32	32		-0.02(-0.55,0.52)	0.005	0.814
HSPA1B	3	32	32	0.17	-1.317(-3.198,0.565)	0.029	0.718
RCN3	3	12	10	0.532	-1.103(-4.561,2.356)	0.003	0.825
TWF2	3	12	10	0.721	-0.322(-2.087,1.443)	0.054	0.658
ENPP1	3	12	10	0.413	0.657(-0.914,2.228)	0.082	0.601
LYPD2	3	33	33	0.185	-0.718(-1.780,0.344)	0.116	0.536
NUCB2	3	12	10	0.447	-1.789(-6.405,2.826)	0.013	0.838
APOA4	3	13	13	0.381	-0.469(-1.518,0.58	0.212	0.356

					0)		
APOH	3	13	13	0.265	-1.238(-3.415,0.938)	0.009	0.79
ITIH2	3	13	13	0.794	-0.105(-0.894,0.684)	0.851	0
PLTP	3	33	33				
IGHM	3	33	33	0.179	1.135(-0.522,2.793)	0.016	0.76
ALDOC	3	11	7	0.221	-1.820(-4.734,1.094)	0.009	0.789
TPM3	3	11	7	0.792	-0.300(-2.528,1.928)	0.021	0.741
TSN	3	11	7	0.631	0.529(-1.633,2.692)	0.013	0.769
HDHD2	3	11	7	0.89	0.278(-3.673,4.229)	0	0.874
C1QBP	3	11	7	0.62	0.713(-2.108,3.533)	0.004	0.816
RPN1	3	11	7	0.931	-0.075(-1.769,1.618)	0.931	0.663
LYPD3	3	11	7	0.077	-1.313(-2.769,0.142)	0.178	0.42
VAT1	3	11	7	0	-2.05(-3.2,-0.91)	0	44.5
NDRG1	3	11	7	0	-2.05(-3.18,-0.93)	0.791	0
LMNB1	3	11	7	0.412	0.861(-1.194,2.917)	0.021	0.742
PURA	3	11	7	0.095	-2.165(-4.707,0.377)	0.048	0.671
ADIRF	3	11	7	0.919	0.200(-3.679,4.079)	0.001	0.862
RAB10	3	11	7	0.589	0.503(-1.318,2.323)	0.035	0.702
PEBP1	3	11	7	0.835	-0.356(-3.713,3.000)	0.001	0.858
LAMP1	3	11	7	0.826	-0.183(-1.813,1.448)	0.076	0.612
ARF4	3	11	7	0.665	-0.530(-2.931,1.870)	0.019	0.749
XRCC6	3	11	7	0.981	0.027(-2.186,2.239	0.01	0.784

					)		
RAB14	3	11	7	0.564	0.751(-1.803,3.306)	0.005	0.814
MRC2	3	11	7	0.017	-1.18[-2.15,-0.21]	0.594	0
PPIB	3	11	7	0.282	-1.009(-2.847,0.829)	0.054	0.657
KERA	3	11	7	0.023	-1.17(-2.18,-0.16)	0.17	43.6
ERP29	3	11	7	0.668	-0.242(-1.349,0.864)	0.002	0.835
NIT2	3	11	7	0.053	1.222(-0.018,2.462)	0.002	0.844
LMNA	3	11	7	0.753	-0.169(-1.219,0.882)	0.006	0.803
DCTN2	3	11	7	0.636	-0.610(-3.134,1.914)	0.01	0.782
GPC1	3	11	7	0.187	-1.017(-2.528,0.493)	0.126	0.517
CBR1	3	11	7	0.927	0.176(-3.584,3.936)	0.003	0.827
ACTN4	3	11	7	0.494	-1.161(-4.490,2.168)	0.002	0.835
TLN1	3	11	7	0.098	-2.305(-5.037,0.426)	0.026	0.727
PDIA3	3	11	7	0.44	-1.100(-3.893,1.693)	0.004	0.82
LSM3	3	11	7	0.822	-0.382(-3.706,2.942)	0.002	0.845
PCBP1	3	11	7	0.598	-1.070(-5.054,2.913	0.001	0.868
LUM	3	11	7	0.492	0.306(-0.569,1.182)	0.806	0
NAMPT	3	11	7	0.85	0.229(-2.140,2.598)	0.009	0.789
PYGB	3	11	7	0.739	-0.464(-3.196,2.268)	0.003	0.833
MTAP	3	11	7	0.548	-1.073(-4.575,2.428)	0.001	0.854
OUTB1	3	11	7	0.777	-0.419(-3.320,2.482)	0.011	0.78
MESD	3	11	7	0.399	-0.729(-2.424,0.966)	0.081	0.602
XRCC5	3	11	7	0.715	-0.518(-3.297,2.262)	0.003	0.83

GPX1	3	11	7	0.786	-0.466(-3.828,2.895)	0.002	0.837
ARL8B	3	11	7	0.243	-1.374(-3.681,0.932)	0.024	0.732
CKAP4	3	11	7	0.605	0.587(-1.636,2.810)	0.011	0.777
CNPY2	3	11	7	0.031	-1.09[-2.09,-0.1]	0.222	33.6
WDR1	3	11	7	0.897	-0.178(-2.868,2.512)	0.005	0.812
CALR	3	11	7	0.988	-0.014(-1.818,1.790)	0.048	0.67
DSG1	3	11	7	0.33	-2.349(-7.073,2.376)	0	0.88
IDH3A	3	11	7	0.365	0.756(-0.878,2.390)	0.064	0.637
DPP3	3	11	7	0.881	-0.213(-3.001,2.576)	0.008	0.794
RACK1	3	11	7	0.599	-0.811(-3.835,2.213)	0.001	0.846
TBCA	3	11	7	0.963	-0.075(-3.188,3.039)	0.002	0.843
PA2G4	3	11	7	0.493	0.792(-1.473,3.057)	0.01	0.784
GSTO1	3	11	7	0.454	0.549(-0.886,1.983)	0.106	0.555
TKT	3	11	7	0.585	-0.551(-2.532,1.430)	0.025	0.728
PARK7	3	11	7	0.412	-0.682(-2.311,0.947)	0.054	0.657
APOA2	3	11	7	0.987	-0.006(-0.672,0.661)	0.28	0.214
HBB	3	11	7	0.853	-0.069(-0.793,0.656)	0.249	0.281
ALB	3	11	7	0.929	-0.043(-0.989,0.902)	0.004	0.735
