# Supplementary material for: The candidate proteins associated with keratoconus: A meta-analysis and bioinformatic analysis
Source: PLoS One. 2024 Mar 14;19(3):e0299739. doi: 10.1371/journal.pone.0299739 (PMC10939257; doi:10.1371/journal.pone.0299739)
Supplement: S6 File — (PDF) [file pone.0299739.s018.pdf]

mRNA	miRNA
LOX	miR-27b-3p
	miR-506-3p
	miR-27a-3p
	miR-30d-5p
	miR-124-3p
	miR-30c-5p
	miR-30e-5p
	miR-24-3p
	miR-3167
	miR-876-5p
VAT1	miR-133a-3p
	miR-506-3p
	miR-3119
	miR-30d-5p
	miR-124-3p
	miR-30c-5p
	miR-30e-5p
	miR-133b
KERA	miR-3167
	miR-876-5p
FMOD	miR-506-3p
	miR-3119
	miR-124-3p
SFRP1	miR-27b-3p
	miR-27a-3p
MRC2	miR-133a-3p
	miR-24-3p
NDRG1	miR-133a-3p
	miR-133b
LYPD3	miR-27b-3p
	miR-27a-3p
FKBP2	miR-133a-3p
